# Loss of the E3 ubiquitin ligase *HACE1* results in enhanced Rac1 signaling contributing to breast cancer progression

**DOI:** 10.1038/onc.2014.468

**Published:** 2015-02-09

**Authors:** E T Goka, M E Lippman

**Affiliations:** 1 Shelia and David Fuente Graduate Program in Cancer Biology, University of Miami Miller School of Medicine, Miami, FL, USA; 2 Department of Medicine, University of Miami Miller School of Medicine, Miami, FL, USA

## Abstract

The transition from ductal carcinoma *in situ* (DCIS) to invasive breast cancer (IBC) is a crucial step in breast cancer progression. The specific alterations that govern this transition have not been elucidated. HER2/*neu* is frequently overexpressed in DCIS but is less common in IBC, thereby suggesting additional requirements for transformation. To identify genes capable of cooperating with HER2/*neu* to fully transform mammary epithelial cells, we used an insertional mutagenesis screen on cells isolated from wild-type *neu* expressing mice and identified the E3 ligase *HACE1* as HER2 cooperative tumor suppressor gene. Loss of *HACE1* expression is commonly seen in clinical breast cancer data sets. *HACE1* downregulation in normal human mammary epithelial cells (HMECs) results in the accumulation of the activated GTP-bound Rac1 partially transforming these cells. Overexpression of HER2 activates Rac1, which further accumulates upon *HACE1* loss resulting in Rac1 hyperactivation. Although the knockdown of *HACE1* or overexpression of HER2 alone in HMECs is not sufficient for tumorigenesis, HER2 overexpression combined with *HACE1* downregulation fully transforms HMECs resulting in robust tumor formation. The pharmaceutical interference of Rac function abrogates the effects of *HACE1* loss both *in vitro* and *in vivo*, resulting in marked reduction in tumor burden. Our work supports a critical role for *HACE1* in breast cancer progression and identifies patients that may benefit from Rac-targeted therapies.

## Introduction

Breast cancer develops through a multistep process driven by genetic and epigenetic changes that gradually transform normal breast epithelium into pre-invasive (pre-cancerous) lesions such as ductal carcinoma *in situ* (DCIS) and finally culminate in invasive breast cancer. Although the transition from DCIS to invasive disease has been implicated as the key transition in breast cancer progression, it is a non-obligate step.^1, 2^ Therefore, the elucidation of alterations that regulate cellular transformation are required to further understand disease progression.

Previous clinical studies identified HER2 (also know as *neu*) overexpression in DCIS lesions as a predictive indicator for the transition of *in situ* to invasive breast cancer.^3^ However, while HER2 overexpression is seen in up to 50% of all DCIS lesions, <25% of invasive breast carcinomas overexpress HER2,^4^ suggesting that HER2 alone is not sufficient for full malignant transformation.

Genetically engineered mice that express the wild-type *neu* proto-oncogene under the control of the mouse mammary tumor virus promoter spontaneously develop focal mammary tumors after a prolonged latency period, suggesting that additional alterations are required for tumorigenesis.^5^ Interbreeding of mouse mammary tumor virus-*neu* mice with other genetically engineered mice models has identified numerous *neu* cooperating genes.^6^ However, this process is both costly and time consuming.

The use of unbiased forward screening approaches has identified novel cancer-associated genes as well as validating known cancer genes. One selection system: validation-based insertional mutagenesis (VBIM), uses a lentiviral-based random insertion of a strong promoter into the genome.^7^ VBIM was used to identify genes capable of cooperating with HER2/neu enabling malignant transformation. We now provide evidence that the loss of HECT and Ankyrin domain containing E3 ubiquitin ligase 1 (*HACE1*), an E3 ligase that tags activated Rac1 for proteosomal degradation, leads to breast cancer transformation. Molecular characterization of *HACE1* in breast cancer shows that *HACE1* attenuates Rac signaling in mammary epithelial cells, and that loss of *HACE1* results in enhanced Rac signaling resulting in tumorigenicity. The pharmaceutical inhibition of Rac diminished the effects of *HACE1* loss-induced transformation. The clinical significance of loss of *HACE1* expression was validated in multiple clinical data sets. Therefore, the ramifications of *HACE1* loss further substantiate the importance of Rac signaling in breast cancer and its role in tumor progression.

## Results

### Insertional mutagenesis screen identifies *HACE1* loss that leads to anchorage-independent growth

**Figure 1:**
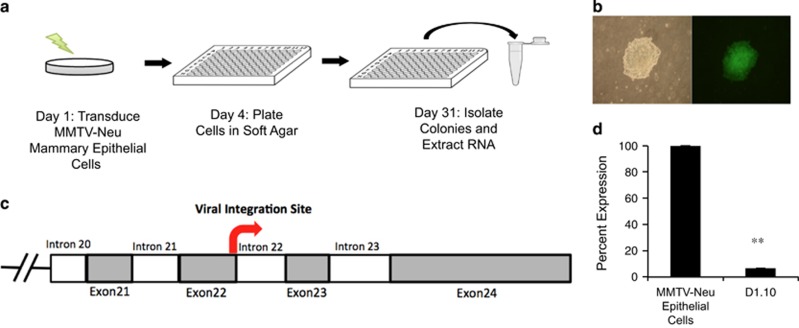
Identification of HACE1 as a HER2/*neu* cooperating tumor suppressor gene. (**a**) VBIM soft agar screen design. (**b**) Typical colony formed in soft agar. Bright-field and green fluorescent protein (GFP) filter images taken at × 10 magnification. (**c**) Mapping of viral integration site of clone 1.10 to murine HACE1 Exon22. (**d**) RT–PCR for HACE1 on cDNA from parental mouse mammary tumor virus-*neu* mouse mammary epithelial cells and clone SD1.10. (**P< 0.001 between groups, Student's *t*-test).

To identify novel contributing factors to breast cancer development and progression, we performed an insertional mutagenesis screen on primary mouse mammary epithelial cells (MMECs) that are partially transformed by expression of the *neu* proto-oncogene under the control of the mouse mammary tumor virus promoter.^5^ At 7 weeks of age, these mice show hyperplasia of their ductal trees indicative of *neu* overexpression. However, isolated MMECs at this time point are unable to form colonies under anchorage-independent conditions. To generate additional genetic events, cells from post-pubescent mice were isolated, transduced with the VBIM lentivirus to generate a library of random transformants^7^ and then screened for anchorage-independent growth (Figure 1a). Colonies that were formed in soft agar were isolated and 3′ RACE was conducted on isolated RNA using gene-specific primers against components of the virus to identify insertion sites. Sequencing results revealed a candidate list of putative oncogenes and tumor suppressor genes, as well as canonical tumor suppressor genes such as caspase-9 and genes shown to be associated with HER2+ breast cancer such as the mitochondrial transporter protein SLC25A43^8^ (Supplementary Table 1). Sequencing of clone 10 (Figure 1b) identified an out-of-frame insertion in Exon 22 of the murine *HACE1*, which has previously been identified as an E3 ubiquitin ligase lost in sporadic Wilms' tumor patients (Figure 1c).^9^ The out-of-frame insertion in clone 10 resulted in a decrease in *HACE1* mRNA expression compared with parental cells (Figure 1d). These data support the notion of *HACE1* as a breast cancer tumor suppressor gene that is capable of cooperating with HER2/neu.

### *HACE1* expression is reduced in human breast tumors

**Figure 2:**
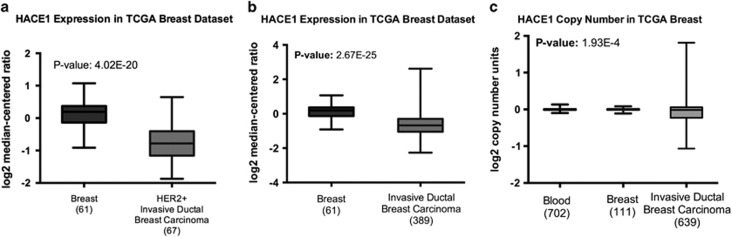
Reduced HACE1 expression in breast cancer data sets. (**a**) mRNA expression analysis for human HACE1 on normal breast and HER2+ invasive ductal breast carcinoma in TCGA breast data set graphed as log2 median-centered ratio. (**b**) mRNA expression analysis for human HACE1 on normal breast and invasive ductal breast carcinoma in TCGA breast data set graphed as log2 median-centered ratio. (**c**) HACE1 DNA copy number for blood, normal breast and invasive ductal breast carcinoma in TCGA breast data set graphed as log2 copy number units. Statistical analyses between normal breast and invasive ductal breast carcinoma were conducted using a Student's *t*-test between groups.

Previous studies have provided evidence that *HACE1* is lost in multiple cancer types due to allelic loss or promoter methylation.^10, 11, 12, 13^ In addition, *HACE1* knockout mice have been shown to develop spontaneous tumors at multiple locations, including the breast, after a prolonged latency period.^13^ *HACE1* expression was analyzed in multiple clinical patient data sets including The Cancer Genome Atlas (TCGA) breast data set. *HACE1* was found to be highly significantly underexpressed in HER2+ invasive ductal breast carcinoma compared with normal breast epithelium (Figure 2a). Interestingly, *HACE1* expression was also highly significantly underexpressed in all invasive ductal breast carcinoma compared with normal breast, suggesting that *HACE1* loss is not confined to HER2 positivity (Figure 2b and Supplementary Figure 1). Moreover, allelic loss of *HACE1* in invasive ductal breast carcinoma was also observed in the TCGA breast data set (Figure 2b). Thus, underexpression of *HACE1* in breast cancer can be attributed to allelic loss of the *HACE1* locus.

To determine whether *HACE1* loss also occurs in other cancer types, we queried *HACE1* expression and/or copy number in multiple clinical data sets. Notably, *HACE1* was underexpressed or underwent allelic loss in cancer compared with respective normal tissues in glioblastoma, melanoma, lymphoma, lung and pancreatic cancers (Supplementary Figure 1). Taken together, these data show that *HACE1* is significantly decreased during the transformation from the normal to malignant state in breast cancer as well as other many other types of cancer.

### *HACE1* knockdown in human mammary epithelial cells leads to transformation

**Figure 3:**
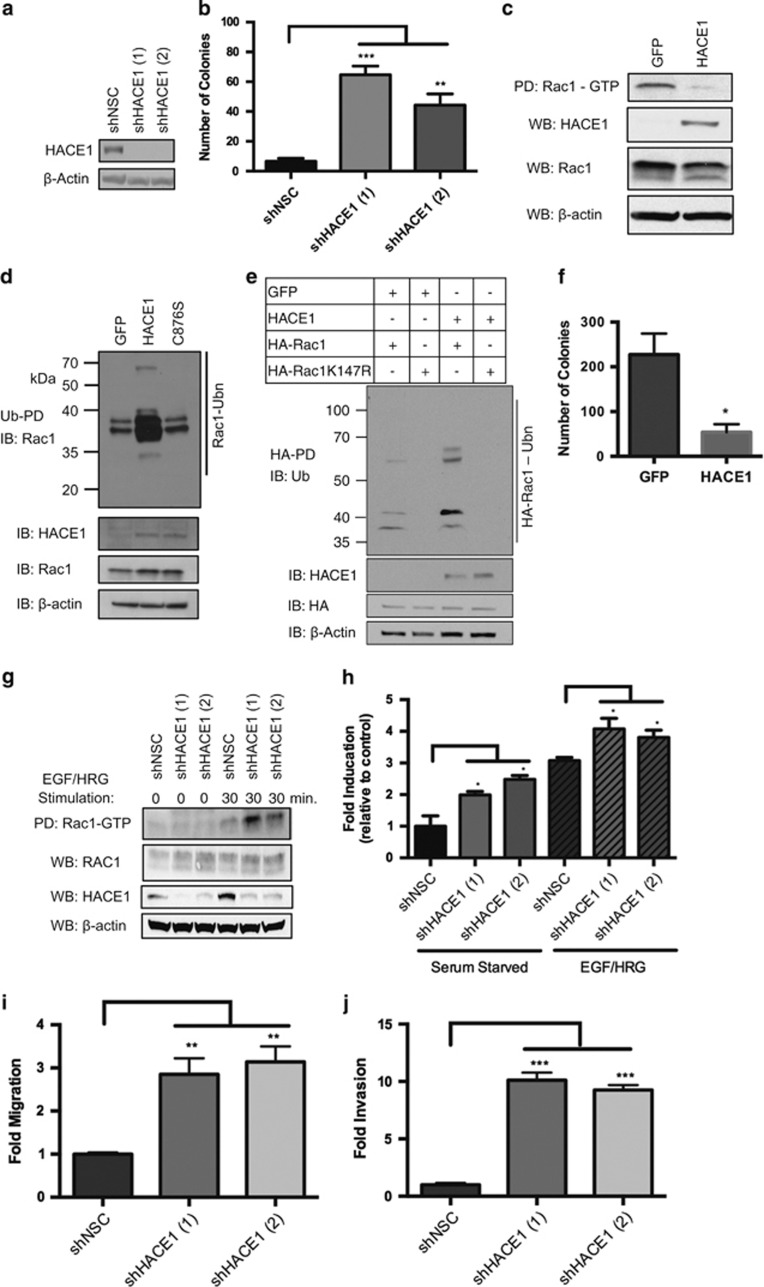
HACE1 controls transformation through regulation of Rac1. (**a**) HACE1 expression in MCF12A cells after treatment with two independent HACE1-specific shRNAs (shHACE1, 1 and 2) as determined by western blotting. Non-silencing control (NSC) shRNA is shown as control. (**b**) Colony formation of MCF12A shHACE1 (1 and 3) and shNSC cells. Data are expressed as mean±s.e.m. of three separate experiments. (**c**) Rac-GTP levels in MCF7 HACE1 overexpressing and GFP control cells stimulated with EGF and HRG as determined by Rac-GTP pull-down. (**d**) Immunoblots show Rac1 ubiquitylation in HRG- and EGF-stimulated MCF7 cells expressing GFP control cells, HACE1 and HACE1 C876S cells. Ub cross-linked forms of Rac1 were purified (Ub-enrichment), resolved on 10% SDS–PAGE and detected by immunoblot for Rac1. (**e**) Immunoblots shows Rac1 ubiquitylation in HRG- and EGF-stimulated MCF7 cells expressing GFP control, HACE1, HA-Rac1 and HA-Rac1K147R. Ubiquitylated HA-Rac was enriched by pull-down, resolved by SDS–PAGE and detected by immunoblot for Ubiquitin. (f) Colony formation of MCF7 GFP control cells and MCF7 HACE1 cells. Data are expressed as mean±s.e.m. of three separate experiments. (**g**) Rac1-GTP levels in MCF12A shHACE1 (1 and 2) knockdown and shNSC cells as determined by Rac1-GTP pull-down. Cells were stimulated for 30 min with 100 ng/ml EGF and 10 ng/ml HRG after overnight starvation. (**h**) Rac1 fold activation of MCF12A shHACE1 (1 and 2) and shNSC cells as determined by Rac1 G-LISA. Cells were stimulated for 30 min with 100 ng/ml EGF and 10 ng/ml HRG after overnight starvation. Data from triplicates (fold increase relative to NSC in the absence of stimuli) are presented as mean±s.e.m. of three independent experiments. (**i**) Migration (Boyden chamber) and (**j**) invasion (modified Boyden chamber) of MCF12A shHACE1 (1 and 2) and shNSC cells (20 h). A quantity of 100 ng/ml EGF and 10 ng/ml HRG was used as chemoattractant between groups, Student's *t*-test). Data are expressed as mean±s.e.m. of three separate experiments. (*P<0.01, **P<0.001, ***P<0.0001 between groups, Student's *t*-test).

To further validate *HACE1* as a human tumor suppressor gene, we determined whether *HACE1* ablation in the normal human mammary epithelial cell line MCF12A increased malignant potential. Two independent short hairpin RNAs (shRNAs) targeting *HACE1* (sh*HACE1*, 1 and 2) and a non-silencing control (shNSC) were introduced into the MCF12A cells (Figure 3a). Transformed cells were plated in soft agar and colony numbers were determined. Although control MCF12A cells were unable to form colonies, cells expressing both shRNAs against *HACE1* showed robust colony formation (Figure 3b). Analysis of *HACE1* ablation in another human mammary epithelial cell line, HME3, recapitulated the results found using the MCF12A cells (Supplementary Figures 2A and B). Thus, *HACE1* knockdown in normal untransformed mammary epithelial cells is sufficient to drive anchorage-independent growth.

### *HACE1* attenuates anchorage-independent growth of human breast cancer cells by reducing levels of activated Rac1

Previous studies have identified the role of ubiquitin-mediated proteosomal degradation of GTP-bound Rac1 in mitigating downstream signaling.^14, 15^ Recent studies identified *HACE1* as the E3 ubiquitin ligase that binds preferentially to GTP-bound Rac1 and catalyzes its polyubiquitylation and subsequent degradation via the proteasome.^16, 17^ Consequently, the loss of *HACE1* resulted in the accumulation of GTP-bound Rac1 resulting in hyperactive Rac signaling. In breast cancer, Rac1 has been reported to be overexpressed and hyperactive.^18^ Upon activation by guanine nucleotide exchange factors (GEFs), active (GTP-bound) Rac1 has been shown to increase cancer cell migration, proliferation, transcription and survival by activating a multitude of downstream effectors.^19^ To determine whether *HACE1* controlled Rac activation in breast cancer cells, *HACE1* was overexpressed in MCF7 breast cancer cell line (Figure 3c). Rac was activated by epidermal growth factor (EGF) and heregulin (HRG), ligands known to potently activate Rac1 in breast cancer cell lines.^20^ Upon activation of Rac1, control MCF7 cells showed potent activation of Rac1 as determined by Rac1-GTP pull-down. Consistent with previous studies, the overexpression of *HACE1* resulted in decreased levels of GTP-bound Rac1 (Figure 3c).^9^ To confirm that the decrease in activated Rac1 levels was due to the polyubiquitation of Rac1 by *HACE1*, we performed ubiquitin precipitation experiments in Rac-stimulated MCF7 cells that overexpress *HACE1*. The overexpression of *HACE1* enhanced Rac1 polyubiquitination compared with control cells, whereas the overexpression of catalytically inactive *HACE1* (C876S) showed no change in levels of Rac1 polyubiquitination (Figure 3d)^16^ Moreover, to verify that *HACE1* ubiquitylation of Rac1 occurs at lysine 147 (citation), we ectopically expressed HA-tagged wild-type Rac1 as well as a K147R mutant. Consistent with previous groups, we observed that *HACE1* overexpression enhances polyubiquitination of wild-type Rac1 but not the K147R mutant (Figure 3d). Thus, the decrease in levels of activated Rac1 in *HACE1* overexpressing MCF7 cells is due to the proteosomal degradation of polyubiquitination of Rac1 at lysine 147 by *HACE1*. Although breast cancer cell lines have reduced levels of *HACE1* compared with their normal counterparts (Supplementary Figure 3), we wished to see whether the reduction of *HACE1* contributes to the their initial transformation. To do so, MCF7 cells that express *HACE1* or control cells were plated in soft agar. Overexpression of *HACE1* in MCF7 breast cancer cells reduced colony formation in soft agar compared with control cells (Figure 3d). These results were recapitulated in another breast cancer cell line, ZR-75-1 (Supplementary Figure 4). Together, these data indicate that breast cancer cells have lost *HACE1* during their transformative process allowing cells to accumulate activated Rac1. *HACE1* expression in breast cancer cell lines can attenuate the levels of activated Rac1 resulting in diminishing their clonogenic potential.

### Loss of *HACE1* leads to accumulation of active Rac signaling in normal mammary epithelial cells

To determine whether *HACE1* loss leads to enhanced Rac1 activity in mammary epithelial cells, MCF12A cells that have *HACE1* knocked down were tested for Rac1 activation. Control and sh*HACE1* knockdown MCF12A cells were serum starved and stimulated with EGF and HRG that induced Rac1 activation (Figure 3e). Knockdown of *HACE1* resulted in enhanced Rac1 activation levels over control cells as determined by Rac1-GTP pull-down. Rac1-GTP levels in *HACE1* knockdown cells were enhanced >2-fold over control cells as determined by Rac1-GTP enzyme-linked immunosorbent assay^21^ corroborating the results of the Rac1-GTP pull-down assays (Figure 3f). Together, these results show that *HACE1* loss in normal mammary epithelial cells results in enhanced levels of Rac1 signaling.

Growth factor-induced Rac activation has long been associated with actin-cytoskeletal rearrangements resulting in enhanced cell motility.^22^ We reasoned that the accumulation of active Rac1 due to *HACE1* loss would result in an increased migratory ability of normal mammary epithelial cells. To investigate whether *HACE1* loss can enhance the migratory ability of normal breast epithelial cells, *HACE1*-depleted MCF12A cells were placed in the upper portion of a Boyden chamber and allowed to migrate through the semi-permeable membrane toward a chemoattractant. *HACE1* knockdown cells showed significant increase in migratory ability (>3-fold) over control cells (Figure 3g and Supplementary Figure 3C). To determine whether *HACE1* loss increases invasiveness (a critical element of the transition from DCIS to invasive breast cancer), *HACE1*-depleted MCF12A cells were plated in the upper portion of a modified Boyden chamber coated with Matrigel. Loss of *HACE1* stimulated the ability of the noninvasive MCF12A cells to invade through Matrigel (Figure 3h). Together, *HACE1* loss in normal mammary epithelial cells leads to elevated levels of Rac activation resulting in enhanced migratory and invasive capabilities.

### Rac1 is required for transformation in *HACE1*-depleted cells

Owing to the exquisite spatial, temporal and substrate specificity requirements for E3 ligases to properly ubiquitylate their target substrates, the number of target substrates is limited. However, E3 ubiquitin ligases have been shown to have multiple target substrates.^23^ Although *HACE1* has been shown to ubiquitylate activated Rac1 for proteosomal degradation, *HACE1* has also been reported to be involved in ubiquitylating a Rab GTPase.^24, 25^ However, Rac1 knockdown in MCF12A cells that had *HACE1* knocked down was capable of reverting their migratory, invasive and clonogenic potential, indicating that the phenotypic effects of *HACE1* loss is driven by the accumulation of activated Rac1 signaling (Supplementary Figure 5). After establishing Rac1 as the major signaling component resulting from *HACE1* loss, we used a small molecule inhibitor EHT1864 known to inhibit Rac by promoting the loss of the nucleotide-bound Rac1 in an inert and inactive state, thus inhibiting downstream signal transduction.^26^

**Figure 4:**
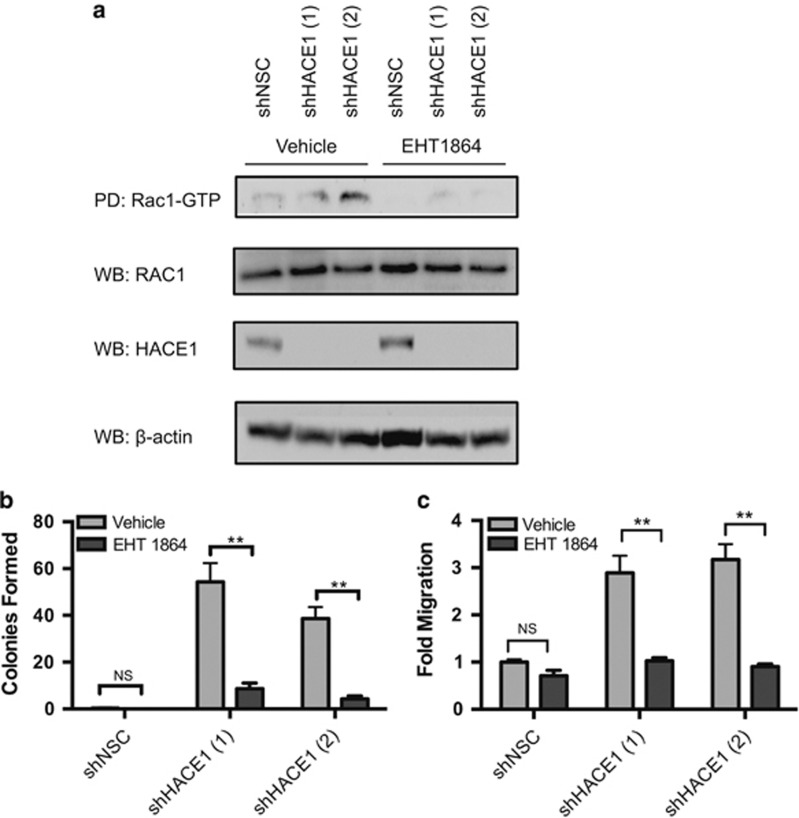
Rac inhibition reverses phenotypic effects of HACE1 loss. (**a**) Rac1-GTP levels in MCF12A shHACE1 (1 and 2) knockdown and MCF12A shNSC cells as determined by Rac1-GTP pull-down. Cells were stimulated for 30 min with 100 ng/ml EGF and 10 ng/ml HRG in the presence of 25 μM EHT1864 or vehicle after overnight serum starvation. (**b**) Soft agar colony formation of MCF12A shHACE1 (1 and 2) and MCF12A shNSC cells in the presence of 50 μM EHT1864 or vehicle (**P<0.001 between groups, Student's *t*-test). Data are expressed as mean±s.e.m. of three separate experiments. (**c**) *In vitro* migration (20 h) of MCF12A shHACE1 (1 and 2) and MCF12A shNSC cells as determined by Boyden chamber in the presence of 25 μM EHT1864 or vehicle. A quantity of 100 ng/ml EGF and 10 ng/ml HRG was used chemoattractant (*P<0.01 between groups, Student's *t*-test). Data are expressed as mean±s.e.m. of three separate experiments.

MCF12A cells were treated with EHT1864 and then stimulated with EGF and HRG to induce Rac1 activation. Pretreatment with the Rac inhibitor EHT1864 at a concentration of 25 μM inhibited Rac signaling in the control MCF12A cells as well as MCF12A cells with *HACE1* knocked down, while the vehicle control had no suppression of Rac1 activation (Figure 4a). These results support previous studies that EHT1864 is a potent RAC inhibitor and indicate that EHT1864 can be used to inhibit Rac1 signaling of human mammary epithelial cells. To rule out nonspecific toxicity, we performed a proliferation and cell viability assay on MCF12A cells. Although EHT1864 caused a marked decrease in cellular proliferation in both control and *HACE1* knockdown cells, cellular viability was unaffected (Supplementary Figure 6).

Just as the knockdown of Rac1 halted anchorage-independent growth (Supplementary Figure 5B), the inhibition of Rac by EHT1864 eliminated the ability of the normal MCF12A cells with *HACE1* knockdown to grow colonies in agar (Figure 4b). These results indicate that EHT1864 blockade of Rac1 signaling is as efficacious as Rac1 ablation by RNA interference in inhibiting the growth of HER2 driven cells as well as the GTP-Rac1 accumulated cell lines.

In order to determine the efficacy of EHT1864 on migration, cells were treated with the Rac inhibitor or vehicle control and plated in a Boyden chamber. Treatment of cells with the Rac inhibitor recapitulated the results of Rac1 knockdown resulting in a twofold reduction in migratory ability (Figure 4c). These results suggest that the inhibition of Rac signaling has the ability to reduce migratory gains caused by *HACE1* loss similar to that of the molecular knockdown of Rac1, and support the mechanism of action of EHT1864 as an inhibitor of Rac signaling.

### HER2 cooperates with *HACE1* loss resulting in accumulation of activated RAC1

**Figure 5:**
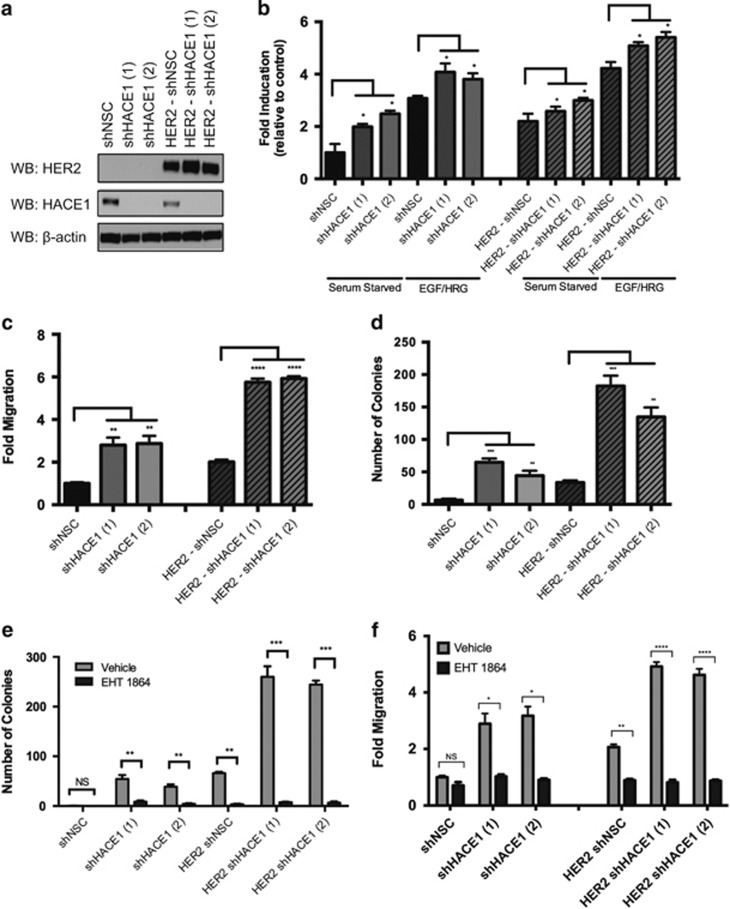
HER2 cooperates with HACE1 to enhance Rac-mediated transformation. (**a)** HACE1 expression in MCF12A cells and MCF12A-HER2 cells after treatment with two independent HACE1-specific shRNAs (shHACE1 (1 and 2) as determined by western blot analysis. Non-silencing control (NSC) shRNA is shown as a control. (**b**) Rac1 fold activation of MCF12A shHACE1 (1 and 2), shNSC, HER2 shHACE1 (1 and 2) and HER2 shNSC cells as determined by Rac1 G-LISA. Cells were stimulated for 30 min with 100 ng/ml EGF and 10 ng/ml HRG after overnight starvation. Data from triplicates (fold increase relative to NSC in the absence of stimuli) are presented as mean±s.e.m. of three independent experiments. (**c**) Migration (20 h) of MCF12A shHACE1 (1 and 2), shNSC, HER2 shHACE1 (1 and 2) and HER2 shNSC cells. A quantity of 100 ng/ml EGF and 10 ng/ml HRG was used as chemoattractant. Data are expressed as mean±s.e.m. of three separate experiments. (**d**) Soft agar colony formation of MCF12A shHACE1 (1 and 2), shNSC, HER2 shHACE1 (1 and 2) and HER2 shNSC cells. Data are expressed as mean±s.e.m. of three separate experiments. (**e**) Colony formation of MCF12A shHACE1 (1 and 2), shNSC, HER2 shHACE1 (1 and 2) and HER2 shNSC cells in the presence of 50 μM EHT1864 or vehicle. Data are expressed as mean±s.e.m. of three separate experiments. (**f**) Migration (20 h) of MCF12A shHACE1 (1 and 2), shNSC, HER2 shHACE1 (1 and 2) and HER2 shNSC cells in the presence of 25 μM EHT1864 or vehicle. A quantity of 100 ng/ml EGF and 10 ng/ml HRG was used as chemoattractant. Data are expressed as mean±s.e.m. of three separate experiments. (*P<0.01, **P<0.001, ***P<0.0001, ****P<0.00001 between groups, Student's *t*-test).

We identified *HACE1* as a tumor suppressor gene that cooperates with HER2. The overexpression of HER2 in normal cells has been previously reported to activate Rac1 by activating RAC1 GEFs such as DOCK1, PREX1 and VAV2.^27, 28, 29, 30^ To determine whether *HACE1* loss would further enhance Rac1 activity when HER2 is overexpressed, MCF12A cells were constructed that stably overexpress HER2 (MCF12A-HER2; Figure 5a). *HACE1* was then stably knocked down as described above. Upon activation of Rac1 using HRG and EGF after serum starvation, potent Rac1 activation was observed in the MCF12A-HER2 control cells as determined by Rac1-GTP enzyme-linked immunosorbent assay. Moreover, *HACE1* knockdown resulted in even greater levels of Rac1 activation than that of the control cells (Figure 5b), suggesting that Rac1 activation by HER2 overexpression cooperates with *HACE1* loss to further enhance Rac1 downstream signaling.

To show that elevated Rac1 activation in the MCF12A-HER2 sh*HACE1* cells correlated with an enhanced cellular phenotype, we performed migration and colony formation assays. HER2 has been previously been shown to enhance migratory and invasive ability of mammary cells through Rac1.^31^ As expected, HER2 overexpression resulted in migratory ability over the control MCF12A cells (Figure 5c). Knockdown of *HACE1* in the MCF12A-HER2 cells resulted in a threefold increase in migration compared with control MCF12A-HER2 cells, suggesting additive effects of HER2 overexpression and *HACE1* loss.

**Figure 6:**
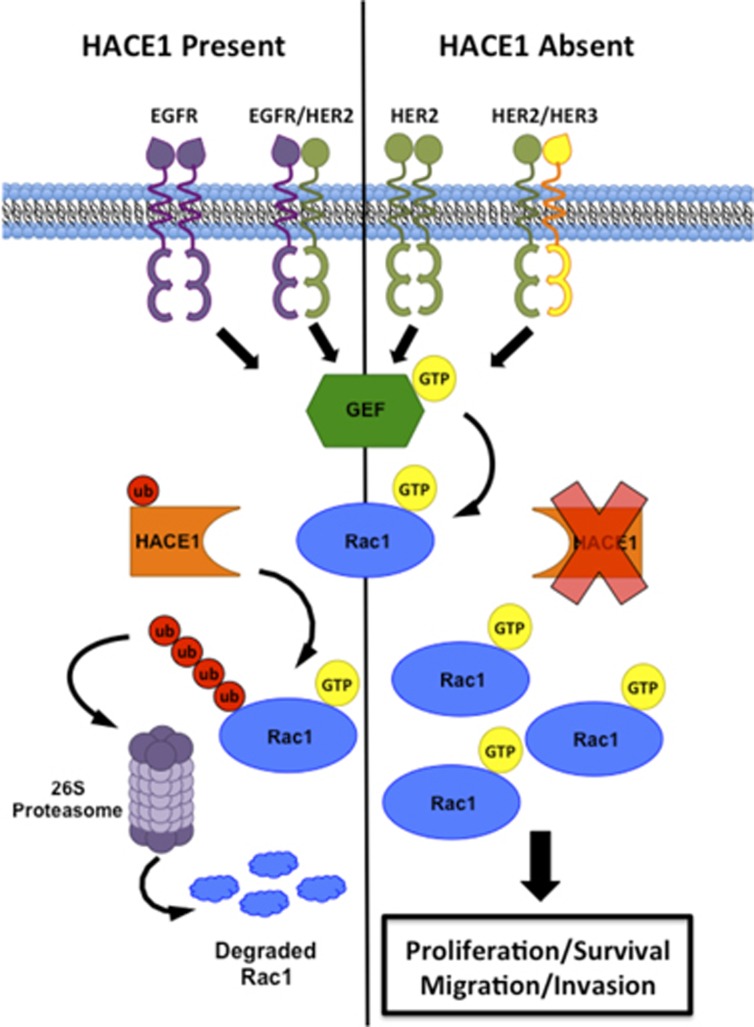
Schematic overview of HACE1 loss in breast cancer. The overexpression of HER2 results in Rac1 activation by homo- or hetero-dimerization of HER family members. Ubiquitylation of Rac1 occurs upon activation when HACE1 is present and Rac1 is degraded via the 26 S proteasome. When HACE1 is lost, activated Rac1 accumulated in the cell driving proliferation, cell survival, migration and invasion.

Although control MCF12A cells were unable to form colonies in soft agar, the overexpression of HER2 in these cells resulted in anchorage-independent growth (Figure 5d). The knockdown of *HACE1* in these MCF12A-HER2 cells resulted in a threefold increase in the number of colonies that were formed, again showing synergy between HER2 and *HACE1* loss. The knockdown of Rac1 using shRNA (Supplementary Figure 7) or pharmaceutical inhibition using the Rac inhibitor EHT1864 reversed the cooperative effects observed by HER2 overexpression and *HACE1* loss. Rac inhibition suppressed colony formation (Figure 5e) as well as migration (Figure 5f), thereby supporting the role of Rac signaling as the key component of transformation. *HACE1* knockdown in a primary HER2+ breast cancer cell line, DT13,^32^ also resulted in enhanced anchorage-independent growth in soft agar owing to enhanced levels of activated Rac1 (Supplementary Figure 8). EHT1864 suppressed the ability of the DT13 cells to grow in soft agar as well as MCF7 cells (Supplementary Figure 9), suggesting that breast cancers including those that overexpress HER2 may be susceptible to Rac-targeted therapies. Therefore, we propose a model in which overexpression of HER2 results in elevated activation of Rac1. When *HACE1* is lost, activated Rac1 accumulates resulting in hyperactive Rac1 signaling (Figure 6).

**Figure 7:**
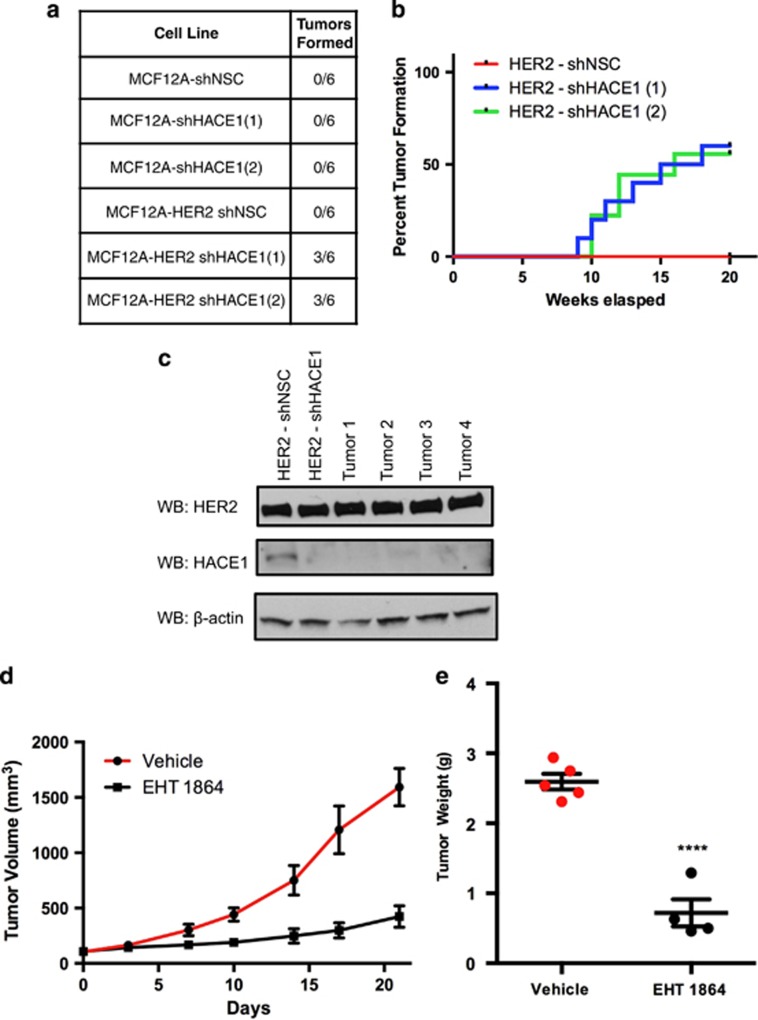
HER2 cooperates with HACE1 allowing full malignant transformation. (**a**) *In vivo* tumor formation of MCF12A shHACE1 (1 and 2), MCF12A shNSC, MCF12A-HER2 shHACE1 (1 and 2) and MCF12A-HER2 shNSC cells. Cells were injected into NOD/SCID mice and allowed to grow for 30 weeks; *n*=6 per group. (**b**) Time course of *In vivo* tumor formation of MCF12A-HER2 shHACE1 (1 and 2) and MCF12A-HER2 shNSC cells. Cells were injected into NOD/SCID mice and allowed to grow for 20 weeks; *n*=10 per group. (**c**) Western blot analysis of MCF12A-HER2 shNSC and shHACE1 cell lines and primary tumors (**d**) MCF12A-HER2 shHACE1 tumor volumes from mice treated with intraperitoneal injections of 30 mg/kg EHT1864 or vehicle. Graphs show mean±s.e.m. of tumor volume at indicated days. *n*=4 or 5 mice per group. (**d**) Graph shows tumor weight after 21 days of vehicle or EHT1864 treatment of MCF12A-HER2 shHACE1 tumors (****P<0.00001 between groups, Student's *t*-test). Graphs show mean±s.e.m. of tumor weight; *n*=4 or 5 mice per group.

To test whether colony formation translates to tumor formation *in vivo*, MCF12A and MCF12A-HER2 cell lines with *HACE1* knockdown were implanted orthotopically into mammary fat pads of NOD-SCID mice. Concordant with our initial hypothesis, the overexpression of HER2 in the MCF12A cells was not enough to allow full malignant transformation (Figure 7a). The MCF12A cells that had enhanced Rac1 activation due to *HACE1* knockdown were also unable to form tumors. Interestingly, only the combination of HER2 and *HACE1* loss were able to generate tumors in (6 out of 12) NOD-SCID mice (Figures 7a and b). These results establish *HACE1* as a tumor suppressor gene that cooperates with HER2 overexpression to fully transform human mammary epithelial cells. HER2 overexpression results in activation of Rac1 signaling, signaling that is then prolonged when *HACE1* is absent. This hyperactivation of Rac1 signaling results in enhanced anchorage-independent growth, migration and tumorigenicity in NOD/SCID mice.

Since our tumor model of HER2 overexpression and *HACE1* loss is driven by Rac1 hyperactivation, we evaluated the antitumor activity of EHT1864 in orthotopic-implanted MCF12A-HER2 sh*HACE1* cells. Mice were pre-randomized to either drug or vehicle control group. Once tumors reached 100 mm^3^ in volume, a bi-weekly treatment regimen began. As shown in Figure 7c, 21 days of EHT1864 treatment significantly reduced tumor size and tumor weight (Figure 7d) relative to vehicle-treated controls while having no obvious toxicity to the mice. These results support the role of Rac in mammary epithelial cell transformation and demonstrate the *in vivo* utilization of the Rac inhibitor EHT1864 to suppress tumor growth.

## Discussion

Over the course of the last few decades, the incidence of DCIS has significantly increased.^33^ This increase results in the overtreatment of patients in whom DCIS will not progress to IDC,^34^ indicating a clear need for predictive markers of disease progression.^35, 36^ HER2/*neu* occurs in up to 50% of *in situ* and only 25% of invasive ductal carcinomas,^37^ suggesting that additional alterations are required to transform DCIS into invasive disease.

Here, using an insertional mutagenesis screen in mammary epithelial cells that express wild-type *neu*, we identify and establish *HACE1* as a breast tumor suppressor gene that attenuates the levels of activated Rac1. We showed that *HACE1* deficiency results in the accumulation of activated Rac1 resulting in enhanced clonogenicty, migration/invasion and tumorigenesis in mice.

We observed that *HACE1* is underexpressed in not only HER2+ breast cancers but also in all human breast cancer patients compared with normal breast epithelium. *HACE1* was found to undergo allelic loss in breast cancer as well as many other cancers while hypermethylation of the *HACE1* promoter has also been observed.^13, 38, 39, 40^ Gene inactivation of Hace1 in mice results in spontaneous cancers after long latency in multiple sites including the breast, further supporting the notion of *HACE1* being a breast tumor suppressor gene.^13^

The tightly regulated balance between active GTP-bound and inactive GDP-bound Rac1 is governed by GEFs and GAPs, respectively. Overexpression of Rac1 GEFs in breast cancer shift the equilibrium toward active GTP-bound Rac1.^19^ Activation of ErbB family members have also been shown to activate Rac1 and its subsequent downstream effectors leading to enhanced mitogenic and motile effects through the activation of GEFs. HER2, in particular, activates numerous Rac GEFs including P-Rex1, VAV2 and DOCK1 resulting in Rac activation in breast cancer.^28, 29, 30^ Constitutively, active Rac1 variants have also been identified in breast cancers such as the splice variant Rac1b^18, 41^ and activating mutational variants,^42^ suggesting that Rac1 signaling is a major component in breast cancer.

In this study, we found that knockdown of *HACE1* in normal breast epithelial cells resulted in enhanced Rac1 activation causing enhanced migratory and invasive ability as well as anchorage-independent growth. Moreover, chronic activation of Rac1 via HER2 overexpression paired with *HACE1* knockdown further enhanced the *in vitro* transformation of these cells. Although knockdown of *HACE1* alone was not sufficient for normal mammary epithelial cells to become tumorigenic in mice, HER2 overexpression coupled with knockdown of *HACE1* allowed robust tumor formation. The ability to identify the active state of Rho GTPases histochemically makes it difficult to identify tumors driven by these Rho GTPases. Hence, the overexpression of upstream transmembrane receptors or Rac GEFs that can either directly or indirectly activate Rac1 combined with *HACE1* loss may identify Rac-driven tumors.

Our findings demonstrate that while sustained Rac1 signaling (due to HER2 overexpression) may be a potent driver of transformation in human breast cancer, *HACE1* is capable of tempering its downstream signaling ability. The Rac inhibitor EHT1864 was able to inhibit Rac1 activation nullifying the phenotypic effects of enhanced Rac signaling *in vitro*.^26, 43^ Although Rac1 knockout mice are embryonic lethal, tissue-specific Rac1 knockout mice are viable, suggesting that Rac1 inhibition in the adult animal may be tolerable.^44^ Our findings using EHT1864 in an *in vivo* setting demonstrate that systemic administration of a Rac inhibitor can be biologically active and may be tolerable to the host, although further studies optimizing dosing and duration is merited. Furthermore, we propose that patients that have both HER2 overexpression combined with *HACE1* loss have hyperactivation of the Rac signaling axis and may benefit from Rac-targeted therapies.

In summary, we performed a screen to identify genes capable of cooperating with HER2 allowing malignant transformation and identified *HACE1* as a breast cancer tumor suppressor gene. We found that *HACE1* loss in mammary epithelial cells and breast cancer leads to enhanced Rac1 signaling resulting in enhanced migration, invasion and anchorage-independent growth. *HACE1* loss coupled with the overexpression of a Rac1 activator such as HER2 results in hyperactivation of Rac signaling and is sufficient to transform normal mammary epithelial cells allowing tumor formation in mice. Because aberrant Rac signaling drives these tumors, treatment with the Rac inhibitor diminishes the oncogenic addiction, thus hauling the Rac-driven phenotypes both *in vitro* and *in vivo*. *HACE1* loss may therefore be utilized as a surrogate for Rac activation, thus identifying patients at risk of disease progression as well as those susceptible to Rac-targeted therapy.

## Materials and methods

### Cell culture and reagents

MCF12A cells (American Type Culture Collection) were grown in DMEM/F12 supplemented with 10 mM HEPES, 10 μg/ml insulin, 20 ng/ml EGF, 20 ng/ml cholera toxin, 30, mM sodium bicarbonate, 0.5 μg/ml hydrocortisone and 5% normal horse serum in a humidified atmosphere containing 5% CO_2_ at 37 °C. MCF7 cells (ATCC) were grown in Improved Minimum Essential Medium (IMEM) (Invitrogen, Carlsbad, CA, USA) with 10% fetal bovine serum. HRG was purchased from R&D Systems (Minneapolis, MN, USA). EGF was purchased from Peprotech (Rocky Hill, NJ, USA). EHT1864 was purchased from Tocris (Minneapolis, MN, USA).

### Oncomine and TCGA analysis

Microarray data from oncomine and TCGA analysis were downloaded for different studies, and expression analysis is plotted as log_2_ median-centered ratio on the *y* axis for gene expression analysis. DNA copy number analysis was plotted as log_2_ copy number units.

### Virus production and infection

VBIM-SD1 was provided by GR Stark (Cleveland Clinic, Cleveland, OH, USA). The GIPZ shRNAs against *HACE1* (shRNA 1: V2LHS_2035241 and shRNA 2: V2LHS_203231) as well as GIPZ shNSC (HS4346) were obtained from Thermo Scientific (Waltham, MA, USA). Viral vectors for *HACE1* (E2838-Lv105), *HACE1* C876S (E2838-C876S-Lv105), HA-Rac1 (EX-A0247-Lv157), control vector (EX-EGFP-Lv105) and HER2 (EX-Z2866-Lv151) were obtained from Genecopoeia (Rockville, MD, USA). HA-Rac1K147R mutant was created using site-directed mutagenesis (QuickChange II, Agilent Technologies, Santa Clara, CA, USA). Lentiviruses were prepared by transfecting 293 T cells with 6 μg plasmid, 4 μg psPAX2 and 2 μg pMD2G using the Lipofectamine 2000 reagent (Invitrogen). Virus-containing supernatants were harvested 48 h after transfection and were used to infect cells.

### Real-time–PCR analysis

mRNA expression was detected by real-time PCR using standard procedures. Primers used include the following: murine *HACE1*, forward 5′-CGTCAACCCTGACTATGCAC-3′ and reverse 5′-CTGCCTGTGGTTCAAAGCTA-3′ and murine GAPDH, forward 5′-ACCCCAGCAAGGACACTGAGCAA-3′ and reverse 5′-TGGGGGTCTGGGATGGAAATTGTG-3′.

### Rac activity assays

Rac1 pull-downs were conducted essentially as previously described (Yang *et al.*^20^). In brief, Rac1 pull-down assays were performed on MCF12A and MCF7 cells. Cells were starved in serum-free media when indicated. Activation was stimulated with 100 ng/ml EGF (Peprotech) and 10 ng/ml HRG (R&D Systems). Activation of Rac1 GTPase was also determined by G-LISA Rac1 colorimetric-based kit (Cytoskeleton, Denver, CO, USA). In brief, MCF12A cells were treated as previously described. When indicated, cells were lysed in the provided lysis buffer and were centrifuged to obtain the soluble extract. After correction of total protein concentration according to the manufacturer's instructions, half of the extract volume (40 μl) was mixed with the provided binding buffer, then added to the enzyme-linked immunosorbent assay plates and incubated on ice for 30 min. Wells were washed and active GTPase was detected by specific antibodies according to the manufacturer's instructions in a microplate spectrophotometer (Bio-Rad, Hercules, CA, USA). An aliquot was also analyzed by western blot analysis to confirm shRNA-mediated *HACE1* depletion. In our hands, the GTPase activation measured in the G-LISA assays was comparable to that obtained in classical Rac1-PBD pull-down assay but with higher sensitivity and thus requiring considerably less cell lysate.

### Western blot analysis

Western blots were conducted as previously described in Yang *et al.*^20^ In brief, cells were lysed with RIPA buffer containing protease inhibitors (Thermo Scientific). Lysates (10–50 μg per lane) were separated by SDS–PAGE, and protein was transferred onto a nitrocellulose membrane (Protran, GE Healthcare Life Sciences, Pittsburgh, PA, USA). The membranes were blocked in 5% milk and were incubated overnight with primary antibodies in 5% bovine serum albumin or milk. Specific antibodies to *HACE1* (1:1000; ab133637, Abcam, Cambridge, MA, USA), HER2/ErbB2 (1:1000; 29D8, Cell Signaling, Danvers, MA, USA), Rac1 (1:500; 05–389, Millipore, Billerica, MA, USA) and actin (1:5,000; ab49900, Abcam) were detected using the appropriate secondary horseradish peroxidase-conjugated antibodies (Bio-Rad) and were visualized by an enhanced chemiluminescence detection system (Pierce, Rockford, IL, USA).

### Ubiquitylation assays

For the enrichment of any ubiquitinated proteins, polyubiquitinated proteins were isolated using high-binding affinity resin (Ubiquitinated Protein Enrichment kit). Cells were serum starved for 16 h and Rac1 activation was achieved using with EGF and HRG while simultaneously being treated with the proteasome inhibitor MG132 (10 μm) for 2 h before cell harvest. Ubiquitin resin was incubated with samples in lysis buffer for 2 h. Resin was then washed four times in the same buffer and bound protein was eluted for SDS–PAGE. The ubiquitinated protein-enriched samples were subjected to immunoblotting for Rac1. For experiments using HA-Rac1, cells were serum starved for 16 h and treated as previously described. Anti-HA agarose (Pierce) was incubated with sample in lysis buffer at 4 °C overnight. Agarose was then washed four times in tris-buffered saline and bound protein was eluted for SDS–PAGE.

### Soft agar assay

Soft agar assays were performed as previously described (Yang *et al.*^20^). In brief, 5 × 10^5^ cells were suspended in 0.6% granulated agar (Sigma, St Louis, MO, USA) diluted in complete medium (× 2) and poured onto a 0.8% layer of agar. Fresh medium was added every 4 days, and 28 days later colonies were stained with crystal violet and counted.

### Transwell migration and invasion assays

Cells were serum starved and 20 000 cells were plated in triplicate into the upper portion of migration or invasion (migration chambers coated with Matrigel) chambers with 8 μM pores (BD Biosciences, San Jose, CA, USA) in 200 μl of serum-free media. A volume of 750 μl of full cell culture medium supplemented with HRG (10 ng/ml) was added to lower chambers as chemoattractant. In experiments using EHT1864, 25 μM EHT1864 or dimethyl sulfoxide was added to media for top and bottom of transwells. After 20 h after plating, media and cells from the upper chambers were removed by aspiration and scrubbing of membranes with cotton swabs. Chambers were fixed in 4% paraformaldehyde for 30 min, washed with phosphate-buffered saline and stained with crystal violet. Five images of the three filters were imaged using a light microscope (× 10 objective) and cells were counted. Data were normalized to migratory counts of control cells and fold migration was calculated. Data were plotted as a fold migration and Student's *t*-tests were used to determine significance.

### Tumor xenograft growth

Experiments involving animals were approved by the Institutional Animal Care and Use Committee of the University of Miami. MCF12A cells were harvested by trypsin digestion and resuspended in phosphate-buffered saline. The MCF12A sh*HACE1* (1 and 2), MCF12A shNSC, MCF12A-HER2 sh*HACE1* (1 and 2) and MCF12A-HER2 shNSC cells were mixed with an equal volume of Matrigel (BD Biosciences). Cell/Matrigel mixture (2 × 10^6^ cells in 100 μl) was inoculated orthotopically into the mammary fat pads of 5-week-old NOD/SCID mice (Jackson Laboratories, Bar Harbor, ME, USA). Tumor size was measured as indicated with a caliper. Tumor size was calculated using the following formula: 0.5 × length × width^2^.

### *In vivo* EHT1864 experiments

Tumors were inoculated into NOD/SCID mice as described above. When tumors reached ~100 mm^3^ in volume, EHT1864 was administered subcutaneously twice a week at a dose of 30 mg/kg body weight (control animals received equal volumes of vehicle, dimethyl sulfoxide). Tumor volume measurements were recorded every 3–4 days until the end point was reached. Tumor size was calculated using the following formula: 0.5 × length × width^2^.

### Statistical analysis

All of the results shown are expressed as means. Standard errors are depicted by error bars in the graphs. All *P*-values were calculated between two groups using Student's *t*-test. *P*<0.05 was considered statistically significant, unless otherwise indicated.

## References

[bib1] 1Wood LD, Parsons DW, Jones S, Lin J, Sjoblom T, Leary RJ et al. The genomic landscapes of human breast and colorectal cancers. Science 2007; 318: 1108–1113. 17932254 10.1126/science.1145720

[bib2] 2Jones PA, Baylin SB. The epigenomics of cancer. Cell 2007; 128: 683–692. 17320506 10.1016/j.cell.2007.01.029 PMC3894624

[bib3] 3Roses RE, Paulson EC, Sharma A, Schueller JE, Nisenbaum H, Weinstein S et al. HER-2/neu overexpression as a predictor for the transition from *in situ* to invasive breast cancer. Cancer Epidemiol Biomarkers Prev 2009; 18: 1386–1389. 19383888 10.1158/1055-9965.EPI-08-1101 PMC2713817

[bib4] 4Latta EK, Tjan S, Parkes RK, O'Malley FP. The role of HER2/neu overexpression/amplification in the progression of ductal carcinoma *in situ* to invasive carcinoma of the breast. Mod Pathol 2002; 15: 1318–1325. 12481013 10.1097/01.MP.0000038462.62634.B1

[bib5] 5Guy CT, Webster MA, Schaller M, Parsons TJ, Cardiff RD, Muller WJ. Expression of the neu protooncogene in the mammary epithelium of transgenic mice induces metastatic disease. Proc Natl Acad Sci USA 1992; 89: 10578–10582. 1359541 10.1073/pnas.89.22.10578 PMC50384

[bib6] 6Marcotte R, Muller WJ. Signal transduction in transgenic mouse models of human breast cancer—implications for human breast cancer. J Mammary Gland Biol Neoplasia. 2008; 13: 323–335. 18651209 10.1007/s10911-008-9087-3

[bib7] 7Lu T, Jackson MW, Singhi AD, Kandel ES, Yang M, Zhang Y et al. Validation-based insertional mutagenesis identifies lysine demethylase FBXL11 as a negative regulator of NFkappaB. Proc Natl Acad Sci USA 2009; 106: 16339–16344. 19805303 10.1073/pnas.0908560106 PMC2736141

[bib8] 8Tina E, Lindqvist BM, Gabrielson M, Lubovac Z, Wegman P, Wingren S. The mitochondrial transporter SLC25A43 is frequently deleted and may influence cell proliferation in HER2-positive breast tumors. BMC Cancer 2012; 12: 350. 22883974 10.1186/1471-2407-12-350 PMC3462703

[bib9] 9Anglesio MS, Evdokimova V, Melnyk N, Zhang L, Fernandez CV, Grundy PE et al. Differential expression of a novel ankyrin containing E3 ubiquitin-protein ligase, Hace1, in sporadic Wilms' tumor versus normal kidney. Hum Mol Genet. 2004; 13: 2061–2074. 15254018 10.1093/hmg/ddh215

[bib10] 10Hibi K, Sakata M, Sakuraba K, Shirahata A, Goto T, Mizukami H et al. Aberrant methylation of the *HACE1* gene is frequently detected in advanced colorectal cancer. Anticancer Res 2008; 28: 1581–1584. 18630515

[bib11] 11Hyytinen ER, Saadut R, Chen C, Paull L, Koivisto PA, Vessella RL et al. Defining the region(s) of deletion at 6q16-q22 in human prostate cancer. Genes Chromosomes Cancer 2002; 34: 306–312. 12007191 10.1002/gcc.10065

[bib12] 12Sakata M, Kitamura YH, Sakuraba K, Goto T, Mizukami H, Saito M et al. Methylation of *HACE1* in gastric carcinoma. Anticancer Res 2009; 29: 2231–2233. 19528486

[bib13] 13Zhang L, Anglesio MS, O'Sullivan M, Zhang F, Yang G, Sarao R et al. The E3 ligase *HACE1* is a critical chromosome 6q21 tumor suppressor involved in multiple cancers. Nat Med 2007; 13: 1060–1069. 17694067 10.1038/nm1621

[bib14] 14Nethe M, Hordijk PL. The role of ubiquitylation and degradation in RhoGTPase signalling. J Cell Sci 2010; 123: 4011–4018. 21084561 10.1242/jcs.078360

[bib15] 15Visvikis O, Maddugoda MP, Lemichez E. Direct modifications of Rho proteins: deconstructing GTPase regulation. Biol Cell 2010; 102: 377–389. 20377524 10.1042/BC20090151

[bib16] 16Torrino S, Visvikis O, Doye A, Boyer L, Stefani C, Munro P et al. The E3 ubiquitin-ligase *HACE1* catalyzes the ubiquitylation of active Rac1. Dev Cell 2011; 21: 959–965. 22036506 10.1016/j.devcel.2011.08.015

[bib17] 17Castillo-Lluva S, Tan CT, Daugaard M, Sorensen PH, Malliri A. The tumour suppressor *HACE1* controls cell migration by regulating Rac1 degradation. Oncogene 2012. 10.1038/onc.2012.189 22614015

[bib18] 18Schnelzer A, Prechtel D, Knaus U, Dehne K, Gerhard M, Graeff H et al. Rac1 in human breast cancer: overexpression, mutation analysis, and characterization of a new isoform, Rac1b. Oncogene 2000; 19: 3013–3020. 10871853 10.1038/sj.onc.1203621

[bib19] 19Wertheimer E, Gutierrez-Uzquiza A, Rosemblit C, Lopez-Haber C, Sosa MS, Kazanietz MG. Rac signaling in breast cancer: a tale of GEFs and GAPs. Cell Signal 2012; 24: 353–362. 21893191 10.1016/j.cellsig.2011.08.011 PMC3312797

[bib20] 20Yang C, Liu Y, Lemmon MA, Kazanietz MG. Essential role for Rac in heregulin beta1 mitogenic signaling: a mechanism that involves epidermal growth factor receptor and is independent of ErbB4. Mol Cell Biol 2006; 26: 831–842. 16428439 10.1128/MCB.26.3.831-842.2006 PMC1347034

[bib21] 21Hernandez E, De La Mota-Peynado A, Dharmawardhane S, Vlaar CP. Novel inhibitors of Rac1 in metastatic breast cancer. P R Health Sci J 2010; 29: 348–356. 21261173

[bib22] 22Ridley AJ, Paterson HF, Johnston CL, Diekmann D, Hall A. The small GTP-binding protein rac regulates growth factor-induced membrane ruffling. Cell 1992; 70: 401–410. 1643658 10.1016/0092-8674(92)90164-8

[bib23] 23Melino G, Gallagher E, Aqeilan RI, Knight R, Peschiaroli A, Rossi M et al. Itch: a HECT-type E3 ligase regulating immunity, skin and cancer. Cell Death Differ 2008; 15: 1103–1112. 18552861 10.1038/cdd.2008.60

[bib24] 24Lachance V, Degrandmaison J, Marois S, Robitaille M, Genier S, Nadeau S et al. Ubiquitylation and activation of a Rab GTPase is promoted by a beta2AR-*HACE1* complex. J Cell Sci 2014; 127(Pt 1): 111–123. 24190883 10.1242/jcs.132944

[bib25] 25Tang D, Xiang Y, De Renzis S, Rink J, Zheng G, Zerial M et al. The ubiquitin ligase *HACE1* regulates Golgi membrane dynamics during the cell cycle. Nat Commun 2011; 2: 501. 21988917 10.1038/ncomms1509 PMC3282116

[bib26] 26Shutes A, Onesto C, Picard V, Leblond B, Schweighoffer F, Der CJ. Specificity and mechanism of action of EHT 1864, a novel small molecule inhibitor of Rac family small GTPases. J Biol Chem 2007; 282: 35666–35678. 17932039 10.1074/jbc.M703571200

[bib27] 27Ueda Y, Wang S, Dumont N, Yi JY, Koh Y, Arteaga CL. Overexpression of HER2 (erbB2) in human breast epithelial cells unmasks transforming growth factor beta-induced cell motility. J Biol Chem 2004; 279: 24505–24513. 15044465 10.1074/jbc.M400081200

[bib28] 28Sosa MS, Lopez-Haber C, Yang C, Wang H, Lemmon MA, Busillo JM et al. Identification of the Rac-GEF P-Rex1 as an essential mediator of ErbB signaling in breast cancer. Mol Cell 2010; 40: 877–892. 21172654 10.1016/j.molcel.2010.11.029 PMC3038344

[bib29] 29Wang SE, Shin I, Wu FY, Friedman DB, Arteaga CL. HER2/Neu (ErbB2) signaling to Rac1-Pak1 is temporally and spatially modulated by transforming growth factor beta. Cancer Res 2006; 66: 9591–9600. 17018616 10.1158/0008-5472.CAN-06-2071

[bib30] 30Laurin M, Huber J, Pelletier A, Houalla T, Park M, Fukui Y et al. Rac-specific guanine nucleotide exchange factor DOCK1 is a critical regulator of HER2-mediated breast cancer metastasis. Proc Natl Acad Sci USA 2013; 110: 7434–7439. 23592719 10.1073/pnas.1213050110 PMC3645577

[bib31] 31Kim IY, Yong HY, Kang KW, Moon A. Overexpression of ErbB2 induces invasion of MCF10A human breast epithelial cells via MMP-9. Cancer Lett 2009; 275: 227–233. 19022565 10.1016/j.canlet.2008.10.013

[bib32] 32Drews-Elger K, Brinkman JA, Miller P, Shah SH, Harrell JC, da Silva TG et al. Primary breast tumor-derived cellular models: characterization of tumorigenic, metastatic, and cancer-associated fibroblasts in dissociated tumor (DT) cultures. Breast Cancer Res Treat 2014; 144: 503–517. 24567196 10.1007/s10549-014-2887-9

[bib33] 33Ernster VL, Barclay J. Increases in ductal carcinoma *in situ* (DCIS) of the breast in relation to mammography: a dilemma. J Natl Cancer Inst Monogr 1997. 151–156. 9709292 10.1093/jncimono/1997.22.151

[bib34] 34Baxter NN, Virnig BA, Durham SB, Tuttle TM. Trends in the treatment of ductal carcinoma *in situ* of the breast. J Natl Cancer Inst 2004; 96: 443–448. 15026469 10.1093/jnci/djh069

[bib35] 35Porter D, Lahti-Domenici J, Keshaviah A, Bae YK, Argani P, Marks J et al. Molecular markers in ductal carcinoma *in situ* of the breast. Mol Cancer Res 2003; 1: 362–375. 12651909

[bib36] 36Ma XJ, Salunga R, Tuggle JT, Gaudet J, Enright E, McQuary P et al. Gene expression profiles of human breast cancer progression. Proc Natl Acad Sci USA. 2003; 100: 5974–5979. 12714683 10.1073/pnas.0931261100 PMC156311

[bib37] 37Barnes DM, Bartkova J, Camplejohn RS, Gullick WJ, Smith PJ, Millis RR. Overexpression of the c-erbB-2 oncoprotein: why does this occur more frequently in ductal carcinoma *in situ* than in invasive mammary carcinoma and is this of prognostic significance? Eur J Cancer 1992; 28: 644–648. 1350456 10.1016/s0959-8049(05)80117-0

[bib38] 38Sakata M, Kitamura YH, Sakuraba K, Goto T, Mizukami H, Saito M et al. Methylation of *HACE1* in gastric carcinoma. Anticancer Res 2009; 29: 2231–2233. 19528486

[bib39] 39Hibi K, Sakata M, Sakuraba K, Shirahata A, Goto T, Mizukami H et al. Aberrant methylation of the *HACE1* gene is frequently detected in advanced colorectal cancer. Anticancer Res 2008; 28: 1581–1584. 18630515

[bib40] 40Sakata M, Yokomizo K, Kitamura Y, Sakuraba K, Shirahata A, Goto T et al. Methylation of the *HACE1* gene is frequently detected in hepatocellular carcinoma. Hepatogastroenterology 2013; 60: 781–783. 23732777 10.5754/hge10439

[bib41] 41Singh A, Karnoub AE, Palmby TR, Lengyel E, Sondek J, Der CJ. Rac1b, a tumor associated, constitutively active Rac1 splice variant, promotes cellular transformation. Oncogene 2004; 23: 9369–9380. 15516977 10.1038/sj.onc.1208182

[bib42] 42Kawazu M, Ueno T, Kontani K, Ogita Y, Ando M, Fukumura K et al. Transforming mutations of RAC guanosine triphosphatases in human cancers. Proc Natl Acad Sci USA 2013; 110: 3029–3034. 23382236 10.1073/pnas.1216141110 PMC3581941

[bib43] 43Onesto C, Shutes A, Picard V, Schweighoffer F, Der CJ. Characterization of EHT 1864, a novel small molecule inhibitor of Rac family small GTPases. Methods Enzymol 2008; 439: 111–129. 18374160 10.1016/S0076-6879(07)00409-0

[bib44] 44Heasman SJ, Ridley AJ. Mammalian Rho GTPases: new insights into their functions from *in vivo* studies. Nat Rev Mol Cell Biol 2008; 9: 690–701. 18719708 10.1038/nrm2476

