# Supplementary material for: Loss of the E3 ubiquitin ligase HACE1 results in enhanced Rac1 signaling contributing to breast cancer progression
Source: Oncogene. 2015 Feb 9;34(42):5395–405. doi: 10.1038/onc.2014.468 (PMC4633721; doi:10.1038/onc.2014.468)
Supplement: Supplementary Table 1 [file onc2014468x1.pdf]

Supplementary Table 1

Putative Oncogene/Tumor Suppressor Gene List
Caspase 9
ASS1
Slc25a43
HACE1
Rragc

Supplementary Table 1 - List of putative oncogenes/tumor suppressor identified in VBIM screen. Genes identified after 3’ RACE on cDNA obtained from VBIM soft agar colonies
