# Supplementary material for: Loss of the E3 ubiquitin ligase HACE1 results in enhanced Rac1 signaling contributing to breast cancer progression
Source: Oncogene. 2015 Feb 9;34(42):5395–405. doi: 10.1038/onc.2014.468 (PMC4633721; doi:10.1038/onc.2014.468)
Supplement: Supplementary Figure 1 [file onc2014468x2.pdf]

Supplementary Fig. 1

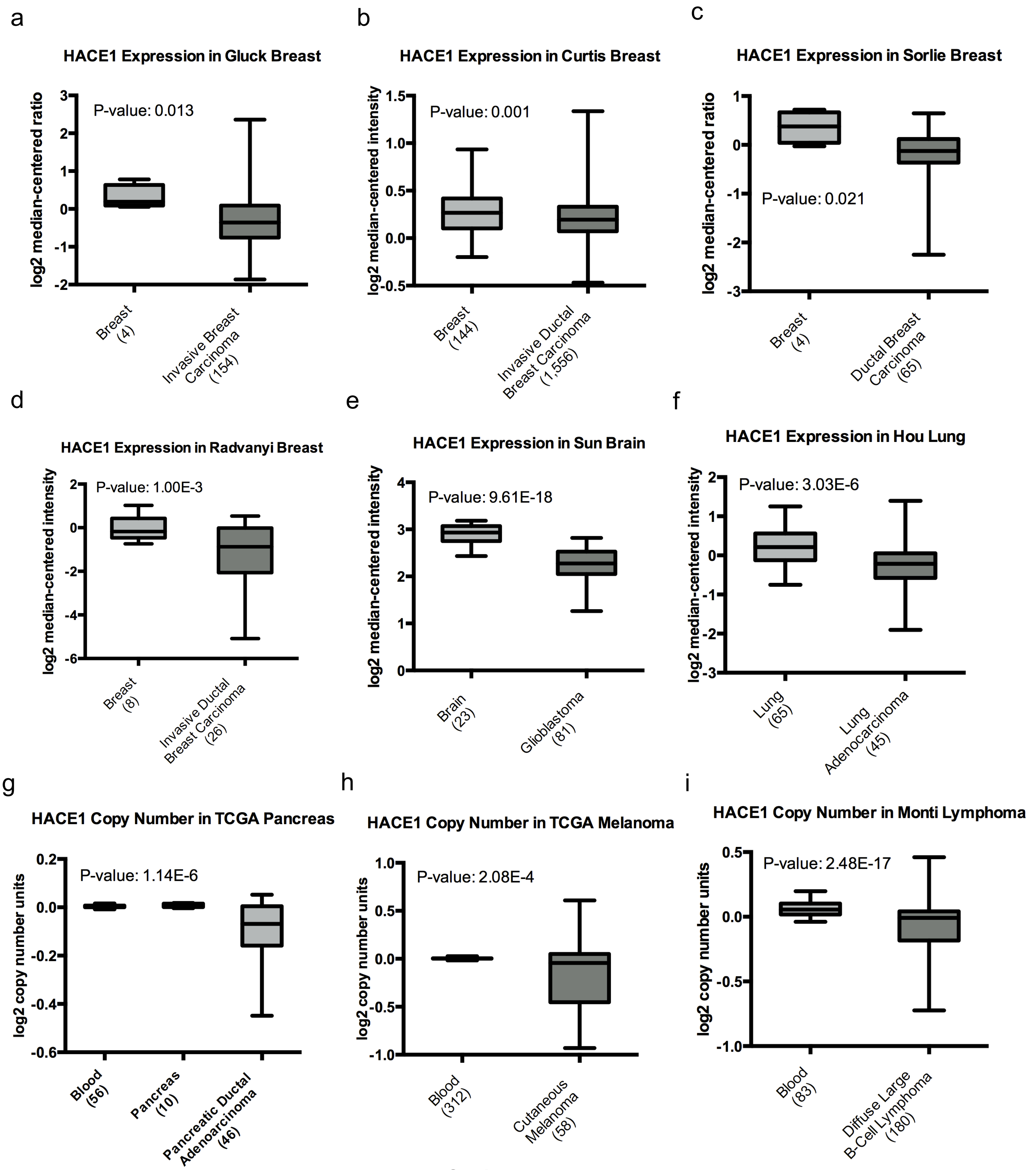

Supplementary Figure 1 – Reduced HACE1 expression in multiple breast cancer datasets. HACE1 mRNA expression in normal breast and breast carcinoma in (a) Gluck Breast, (b) Curtis Breast, (c) Sorlie Breast, and (d) Radvanyi Breast clinical datasets. HACE1 mRNA expression in (E) normal brain and glioblastoma, (F) normal lung and lung adenocarcinoma. HACE1 DNA copy number in(G) blood, normal pancreas, and pancreatic ductal adenocarcinoma, (H) blood and cutaneous melanoma, and (I) blood and diffuse large B-Cell lymphoma. All figures are modified from Oncomine™ (Compendia Bioscience, Ann Arbor, MI) was used for analysis and visualization. P-value determined by Student’s t-test.
