# Supplementary material for: Loss of the E3 ubiquitin ligase HACE1 results in enhanced Rac1 signaling contributing to breast cancer progression
Source: Oncogene. 2015 Feb 9;34(42):5395–405. doi: 10.1038/onc.2014.468 (PMC4633721; doi:10.1038/onc.2014.468)
Supplement: Supplementary Figure 2 [file onc2014468x3.pdf]

Supplementary Fig. 2

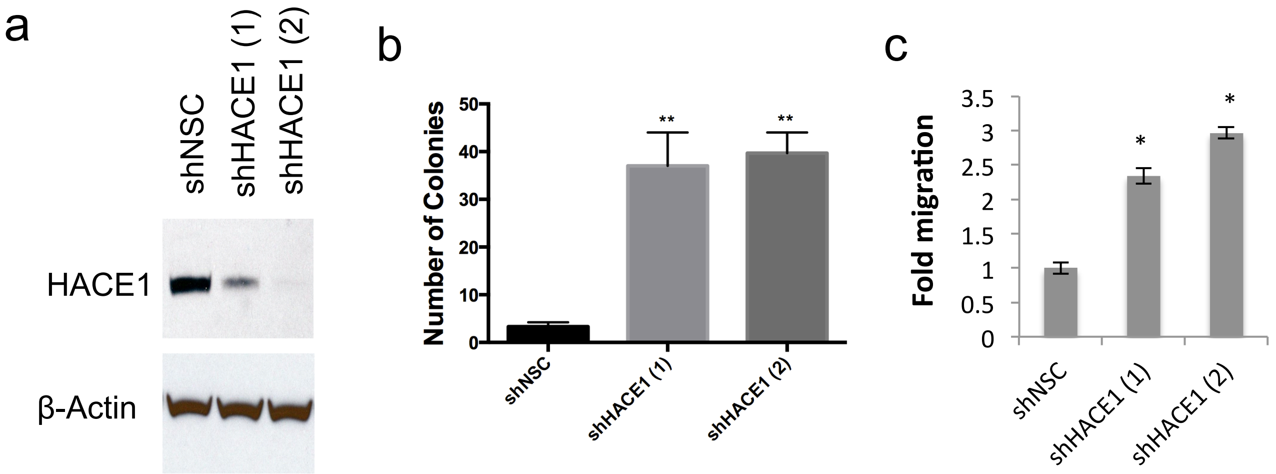

Supplementary Fig. 2 – HACE1 knockdown transforms normal like HME3 cells. (**a**) Decreased HACE1 expression in HME3 cells after treatment with two independent HACE1-specific shRNAs (shHACE1 (1) and shHACE1 (2)) as determined by western blot analysis. Non-silencing control (NSC) shRNA is shown as a control. (**b**) Soft agar colony formation of HME3 shHACE1 (1), HME3 shHACE1 (2), and HME3 shNSC cells. (**P < 0.001 between groups, Student's t-test). Data are expressed as mean ± SEM of three separate experiments. (**c**) *in vitro* migration (20 h) of HME3 shHACE1 (1 & 2) and HME3 shNSC cells as determined by Boyden chamber. 100 ng/ml EGF and 10 ng/ml HRG was used chemotractant (**P < 0.001 between groups, Student's t-test).
