# Supplementary material for: Loss of the E3 ubiquitin ligase HACE1 results in enhanced Rac1 signaling contributing to breast cancer progression
Source: Oncogene. 2015 Feb 9;34(42):5395–405. doi: 10.1038/onc.2014.468 (PMC4633721; doi:10.1038/onc.2014.468)
Supplement: Supplementary Figure 3 [file onc2014468x4.pdf]

Supplementary Fig. 3

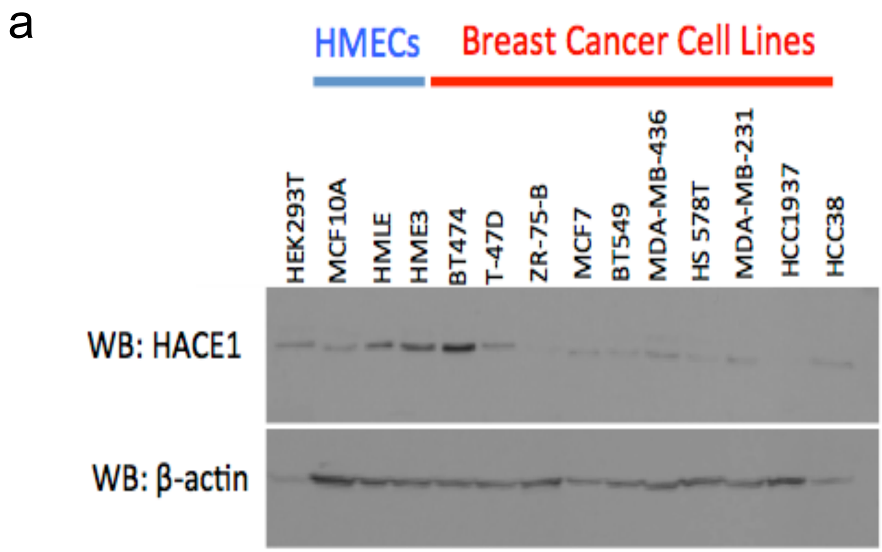

Supplementary Fig. 3 – HACE1 is down in breast cancer cell lines. (a) Western blot analysis for HACE1 on a panel of human mammary epithelial cells (HMECs) and established breast cancer cell lines. Actin is used as control.
