# Supplementary material for: Loss of the E3 ubiquitin ligase HACE1 results in enhanced Rac1 signaling contributing to breast cancer progression
Source: Oncogene. 2015 Feb 9;34(42):5395–405. doi: 10.1038/onc.2014.468 (PMC4633721; doi:10.1038/onc.2014.468)
Supplement: Supplementary Figure 4 [file onc2014468x5.pdf]

Supplementary Fig. 4

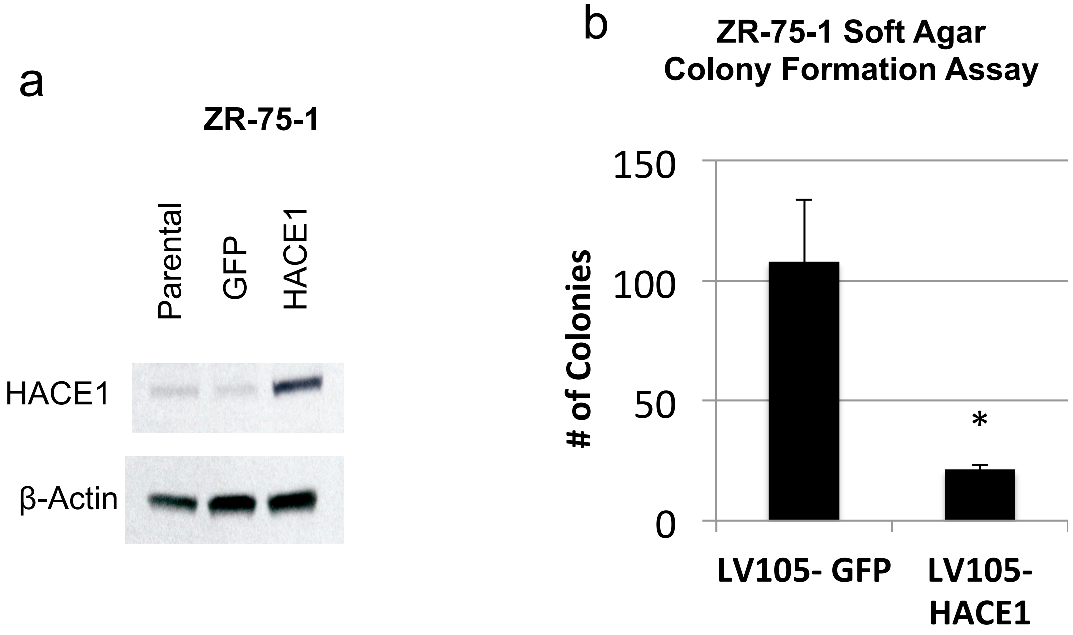

Supplementary Fig. 4 – HACE1 expression in ZR-75-1 cells reduces clonogenicity (a) stable HACE1 overexpression in ZR-75-1 determined by western blot. GFP is control. (**b**) Soft agar colony formation of ZR-75-1 HACE1 and GFP control cells. (1), (*$P < 0.01$ between groups, Student's t-test). Data are expressed as mean ± SEM of three separate experiments.
