# Supplementary material for: Loss of the E3 ubiquitin ligase HACE1 results in enhanced Rac1 signaling contributing to breast cancer progression
Source: Oncogene. 2015 Feb 9;34(42):5395–405. doi: 10.1038/onc.2014.468 (PMC4633721; doi:10.1038/onc.2014.468)
Supplement: Supplementary Figure 5 [file onc2014468x6.pdf]

Supplementary Fig. 5 – Rac1 shRNA knockdown reverses the effects of HACE1 loss

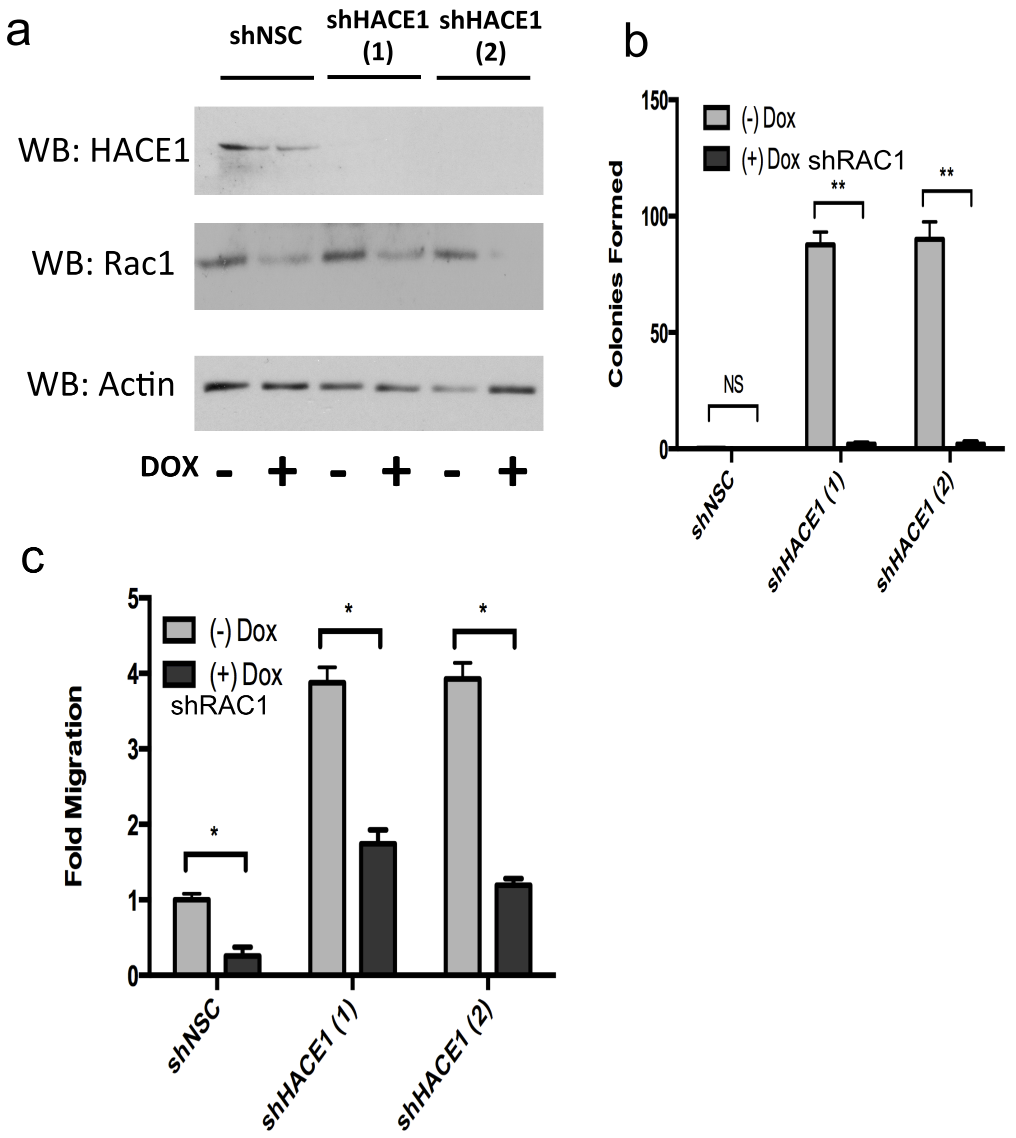

Supplementary Fig. 5 – Rac1 shRNA knockdown reverses the effects of HACE1 loss
(a) Decreased Rac1 after Doxycycline (DOX) induction of Rac1 shRNA in MCF12A-HACE1 knockdown cells (shHACE1 (1) and shHACE1 (2)) and MCF12A shNSC cells. (**b**) Soft agar colony formation of MCF12A shHACE1 (1), MCF12A shHACE1 (2), and MCF12A shNSC cells with and without DOX induction of Rac1 shRNA (**P < 0.001 between groups, Student's t-test). Data are expressed as mean ± SEM of three separate experiments.(**c**) *in vitro* migration (20 h) of MCF12A shHACE1 (1 & 2) and MCF12A shNSC cells with and without DOX induction of Rac1 shRNA as determined by Boyden chamber. 100 ng/ml EGF and 10 ng/ml HRG was used chemotractant (*P < 0.01 between groups, Student's t-test).
