# Supplementary material for: Loss of the E3 ubiquitin ligase HACE1 results in enhanced Rac1 signaling contributing to breast cancer progression
Source: Oncogene. 2015 Feb 9;34(42):5395–405. doi: 10.1038/onc.2014.468 (PMC4633721; doi:10.1038/onc.2014.468)
Supplement: Supplementary Figure 6 [file onc2014468x7.pdf]

Supplementary Fig. 6

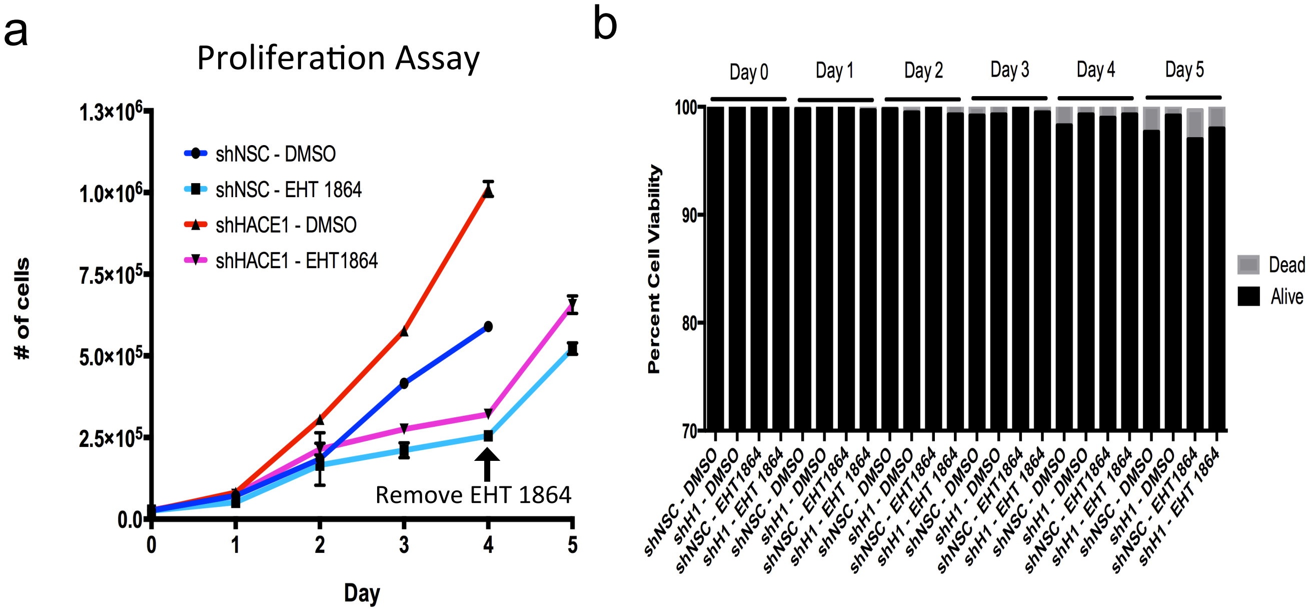

Supplementary Fig. 6 – Long term EHT 1864 treatment reduces cell proliferation while not effecting cell viability (a) Cell counts for MCF12A shHACE1 and MCF12A shNSC cells in the presence of EHT1864 (50 µg/ml) or vehicle (DMSO). EHT 1864 was removed from MCF12A shHACE1 and MCF12A shNSC cells treated with EHT 1864 on day 4. (b) Viability of MCF12A shHACE1 and MCF12A shNSC cells in the presence of of EHT1864 (50 µg/ml) or vehicle (DMSO) determined by Trypan blue exclusion.
