# Supplementary material for: Loss of the E3 ubiquitin ligase HACE1 results in enhanced Rac1 signaling contributing to breast cancer progression
Source: Oncogene. 2015 Feb 9;34(42):5395–405. doi: 10.1038/onc.2014.468 (PMC4633721; doi:10.1038/onc.2014.468)
Supplement: Supplementary Figure 7 [file onc2014468x8.pdf]

Supplementary Fig. 7

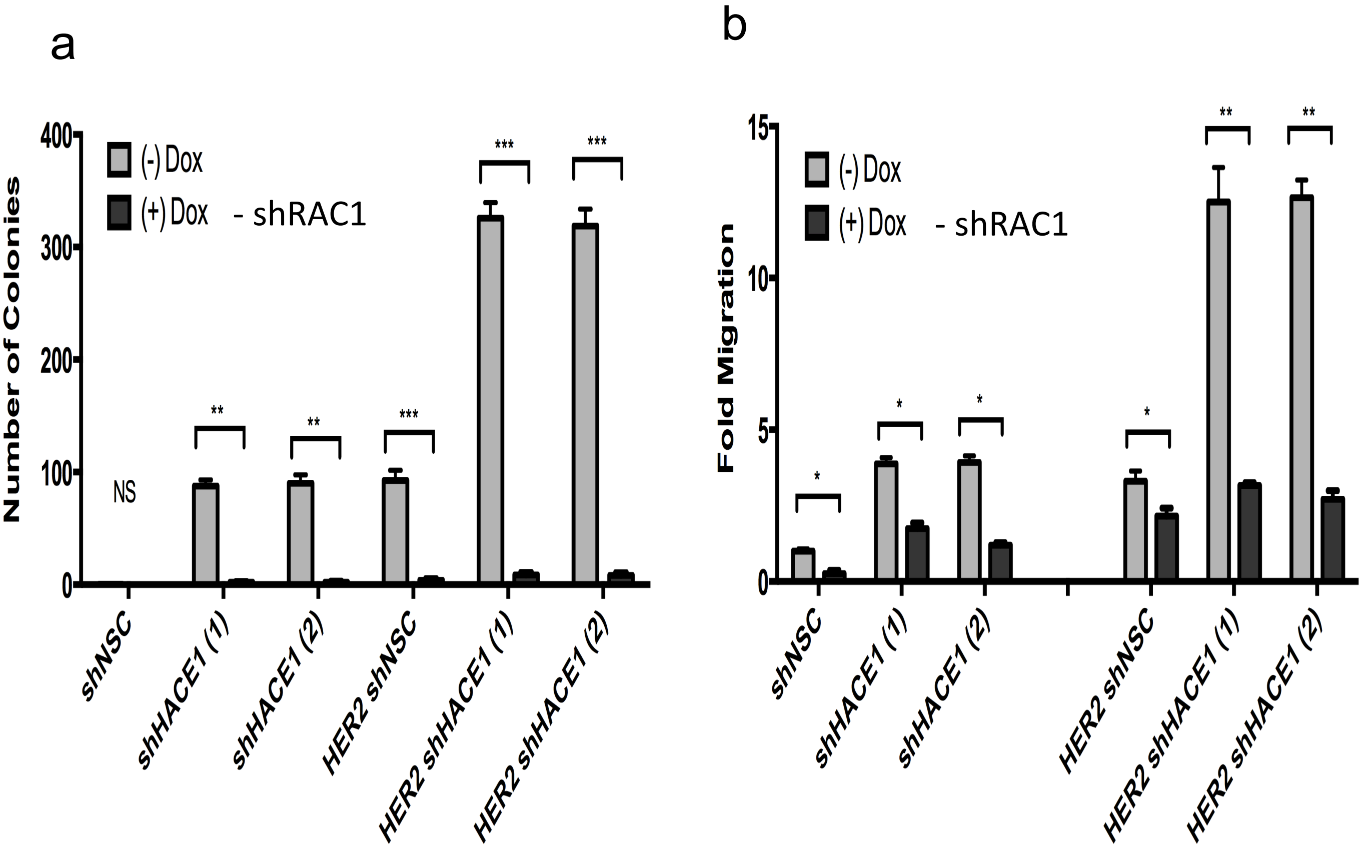

Supplementary Fig. 7 – Rac1 shRNA knockdown reverses cooperative effects of HER2 overexpression and HACE1 loss (a) Soft agar colony formation of MCF12A shHACE1 (1), MCF12A shHACE1 (2), MCF12A shNSC, MCF12A-HER2 shHACE1 (1), MCF12A-HER2 shHACE1 (2), MCF12A-HER2 shNSC cells with and without DOX induction of Rac1 shRNA (**$P < 0.001$, ***$P < 0.0001$ between groups, Student's t-test). Data are expressed as mean ± SEM of three separate experiments. (**b**) *in vitro* migration (20 h) of MCF12A shHACE1 (1), MCF12A shHACE1 (2), MCF12A shNSC, MCF12A-HER2 shHACE1 (1), MCF12A-HER2 shHACE1 (2), MCF12A-HER2 shNSC cells with and without DOX induction of Rac1 shRNA as determined by Boyden chamber. 100 ng/ml EGF and 10 ng/ml HRG was used chemotractant (*$P < 0.01$, **$P < 0.001$ between groups, Student's t-test). Data are expressed as mean ± SEM of three separate experiments.
