# Supplementary material for: Loss of the E3 ubiquitin ligase HACE1 results in enhanced Rac1 signaling contributing to breast cancer progression
Source: Oncogene. 2015 Feb 9;34(42):5395–405. doi: 10.1038/onc.2014.468 (PMC4633721; doi:10.1038/onc.2014.468)
Supplement: Supplementary Figure 8 [file onc2014468x9.pdf]

Supplementary Fig. 8

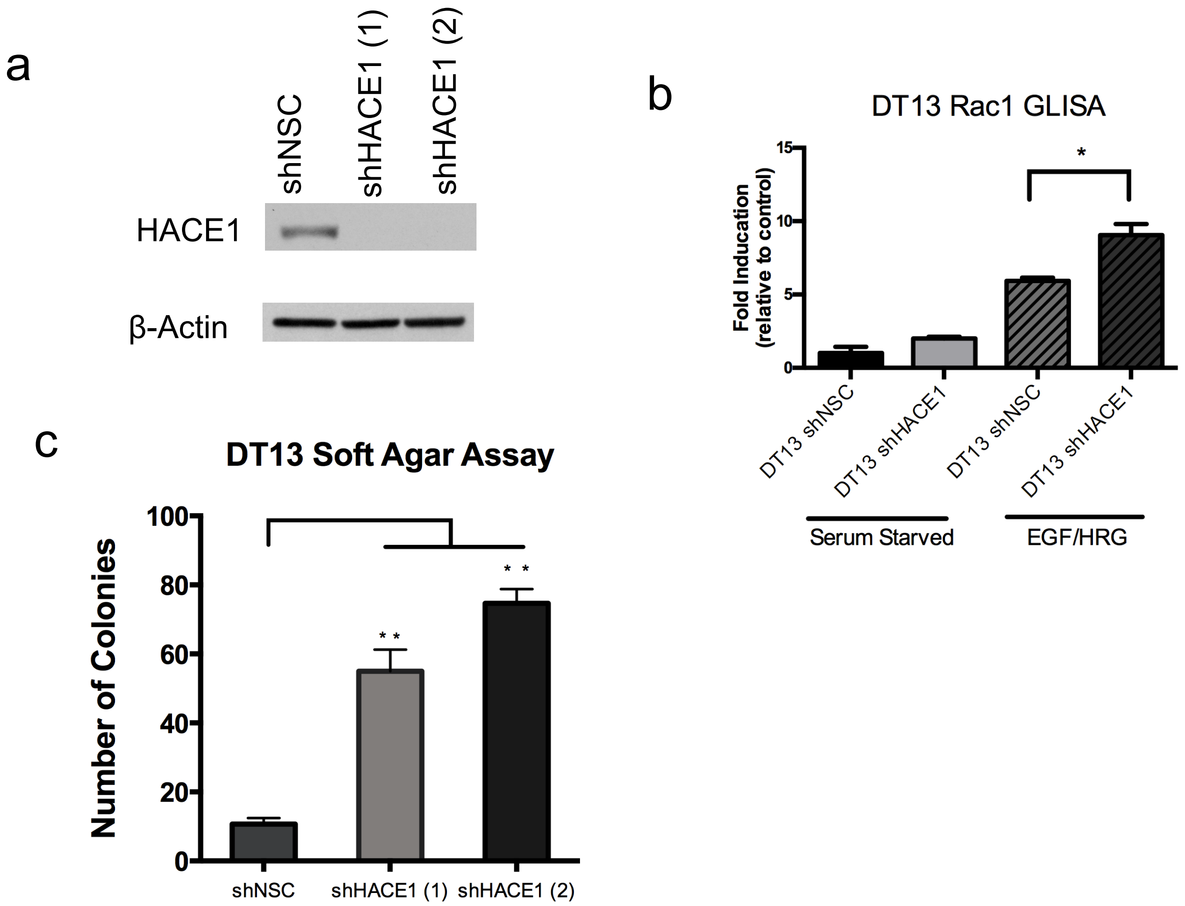

Supplementary Fig. 8 – Knockdown of HACE1 enhances Rac activation of DT13 primary breast cancer cells resulting in enhanced clonogenicity (a) Decreased HACE1 expression in DT13 primary breast cancer cells after treatment with two independent HACE1-specific shRNAs (shHACE1 (1) and shHACE1 (2)) as determined by western blot analysis. **(b)** Activated Rac1 levels determined by Rac1 GLISA of control DT13 and shHACE1 knockdown cells pre and post stimulation with EGF and HRG. Data are presented as fold change compared to serum starved control DT13 cells. (*$P < 0.05$ between groups, Student's t-test). (**c**) Soft agar colony formation assay of control DT13 cells and shHACE1 knockdown cells. (**$P < 0.005$ between groups, Student's t-test).
