# Supplementary material for: Loss of the E3 ubiquitin ligase HACE1 results in enhanced Rac1 signaling contributing to breast cancer progression
Source: Oncogene. 2015 Feb 9;34(42):5395–405. doi: 10.1038/onc.2014.468 (PMC4633721; doi:10.1038/onc.2014.468)
Supplement: Supplementary Figure 9 [file onc2014468x10.pdf]

Supplementary Fig. 9

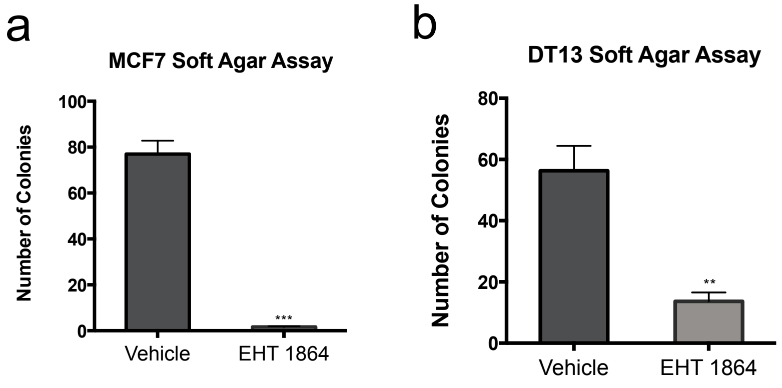

Supplementary Fig. 9 – Rac1 inhibition attenuates growth of breast cancer cell lines in soft agar (a) Soft agar colony formation of MCF7 cells treated with vehicle or 25 μM EHT 1864 (***$P < 0.0001$ between groups, Student's t-test). (**b**) Soft agar colony formation of DT13 cells treated with vehicle or 25 μM EHT 1864 (**$P < 0.001$, between groups, Student's t-test). Data are expressed as mean ± SEM of three separate experiments.
