# Supplementary material for: Loss of the E3 ubiquitin ligase HACE1 results in enhanced Rac1 signaling contributing to breast cancer progression
Source: Oncogene. 2015 Feb 9;34(42):5395–405. doi: 10.1038/onc.2014.468 (PMC4633721; doi:10.1038/onc.2014.468)
Supplement: Supplementary Information 1 [file onc2014468x11.doc]

**Supplemental Methods**

**Identification of VBIM insertion sites from RNA obtained from soft agar colonies.** RNA from soft agar colonies were isolated (RNeasy Kit, Qiagen) and converted to cDNA using oligodT20 as the primer (Superscript III, Invitrogen). 3’ Rapid Amplification of cDNA ends (RACE) was performed using forward primers against the provirus (VBIM for 1: CAACCCACAAGGAGACGACCT, VBIM for 2: CGTTCGTCCTCACTCTCTTCC, VBIM for 3: CGTTCGTCCTCACTCTCTTCC) and an oligodT20 as the reverse primer using taq polymerase (Promega). PCR products were gel extracted (Gel extraction kit, Qiagen) and TA cloned into shuttle plasmid (pCR 2.1, Invitrogen). Plasmids were transformed into DH5α competent cells and grown in LB broth (Invitrogen). DNA miniprep (Qiagen) was performed to obtain DNA and sent for sequencing (University of Miami Oncogenomics Core Facility). BLAT search was performed against the mouse genome (UCSC Genome Bioinformatics Site) to identify insertional sites.

**Isolation and culture of mouse mammary epithelial cells**. Primary mouse mammary epithelial cells were prepared as described previously . Briefly, mammary fat pads were minced, digested with collagenase type II (Invitrogen), passed through a 100 μm pore size cell strainer (BD Biosciences) and the cell suspension plated in 6 – well plates (Greiner bio-one). Cells were cultured routinely in a 50:50 mix HAMs F12 and DMEM media supplemented with 10% FBS, 10 mM HEPES (Invitrogen), 10 ng/ml mouse EGF (Sigma-Aldrich), 1 mg/ml Hydrocortisone (Sigma-Aldrich), 5 μg/ml insulin (Sigma-Aldrich), and antibiotic at 37°C and 5 % CO2.

***Cell lines and reagents.*** HME3 and HMLE3 cells (Obtained from Tan Ince, University of Miami, Miami, FL, USA) were grown in a humidified atmosphere containing 5% CO_2_ at 37°C in MEGM Mammary Epithelial Cell Growth Medium (Lonza), supplemented with bovine pituitary extract (52 μg/mL), hydrocortisone (0.5μg/mL), human EGF (10 ng/mL), and insulin (5 μg/mL). DT13 primary breast cancer cell were generated as previously described were grown in in a humidified atmosphere containing 5% CO_2_ at 37°C in Improved MEM (IMEM, Invitrogen) with 10% fetal bovine serum. MCF-10A cells were obtained from ATCC and grown in DMEM/F12 supplemented with 10mM HEPES, 10 μg/ml insulin, 20 ng/ml EGF, 100 ng/ml cholera toxin, 30, mM sodium bicarbonate, 0.5 μg/ml hydrocortisone, and 5% normal horse serum. Breast cancer cell lines MCF7, T47D, MDA-MB-361, MDA-MB-231, HS578T, HCC1937 and HCC38 were grown in Improved MEM (IMEM, Invitrogen) with 10% fetal bovine serum in a humidified atmosphere containing 5% CO_2_ at 37°C. Breast Cancer cell lines BT474, BT549, and ZR-75-1 were grown in RPMI (with l-glutamine; Invitrogen) with 10% fetal bovine serum in a humidified atmosphere containing 5% CO_2_ at 37°C. Dox-inducible TRIPZ lentiviral Rac1 shRNA clone (V2THS_202655) was obtained from Open Biosystems. Lentiviruses were prepared by transfecting 293T cells with 6 µg TRIPZ plasmid, 4 µg psPAX2, and 2 µg pMD2G using the Lipofectamine 2000 reagent (Invitrogen, Carlsbad, CA). Virus-containing supernatants were harvested 48 hours after transfection and used to infect MCF12A shHACE1 (1 & 2), MCF12A shNSC, MCF12A-HER2 shHACE1 (1 & 2), and MCF12A-HER2 shNSC cells.

**Cell proliferation assay.** Cells were plated 6 well plates in 1 ml of culture medium. EHT 1864 (50 μM) or vehicle (DMSO) were added 24 hours after cell plating. At the time indicated in the figure legends, cells were trypsinized and counted using a hemocytometer. On day 4, EHT 1864 was washed out by removing drug containing media, washing twice with PBS and adding media without drug.

**Cell viability assay.** Trypan blue exclusion was used to determine cell viability as previously described . Briefly, cells were plated 6 well plates in 1 ml of culture medium. EHT 1864 (50 μM) or vehicle (DMSO) were added 24 hours after cell plating. At the time indicated in the figure legends, cells were stained in 0.4% Trypan Blue (Sigma-Aldrich) and counted. Data are represented as percentage of viable cells.

**Supplemental References**

1. Smalley, M.J., *Isolation, culture and analysis of mouse mammary epithelial cells.* Methods Mol Biol, 2010. **633**: p. 139-70.

2. Ince, T.A., A.L. Richardson, G.W. Bell, M. Saitoh, S. Godar, A.E. Karnoub, J.D. Iglehart, and R.A. Weinberg, *Transformation of different human breast epithelial cell types leads to distinct tumor phenotypes.* Cancer Cell, 2007. **12**(2): p. 160-70.

3. Drews-Elger, K., J.A. Brinkman, P. Miller, S.H. Shah, J.C. Harrell, T.G. da Silva, Z. Ao, A. Schlater, D.J. Azzam, K. Diehl, D. Thomas, J.M. Slingerland, C.M. Perou, M.E. Lippman, and D. El-Ashry, *Primary breast tumor-derived cellular models: characterization of tumorigenic, metastatic, and cancer-associated fibroblasts in dissociated tumor (DT) cultures.* Breast Cancer Res Treat, 2014. **144**(3): p. 503-17.

4. Strober, W., *Trypan blue exclusion test of cell viability.* Curr Protoc Immunol, 2001. **Appendix 3**: p. Appendix 3B.
